# Multi-Strategy Improved Red-Billed Blue Magpie Optimization Algorithm and Its Engineering Applications

**DOI:** 10.3390/biomimetics11040287

**Published:** 2026-04-21

**Authors:** Junchao Ni, Jianhua Miao, Yejun Zheng, Li Cao, Yang Qiu, Yinggao Yue

**Affiliations:** 1 School of Electronic and Electrical Engineering, Wenzhou University of Technology, Wenzhou 325035, China; 2 Shanghai Caoyang Vocational School, Shanghai 200333, China; 3 Department of Engineering Technology Education, Shanghai Caoyang Vocational School, Shanghai 200333, China

**Keywords:** red billed blue magpie optimizer, swarm intelligence optimization, chaotic mapping, Lévy flight, differential mutation

## Abstract

In response to the decline in population diversity, the imbalance between exploration and exploitation, and the low convergence efficiency in the middle and later stages of the Red-billed Blue Magpie Optimizer (RBMO) when addressing complex optimization problems, this study proposes a multi-strategy enhanced variant termed CLD-RBMO. The proposed algorithm improves the original search mechanism from three perspectives: strengthened global exploration, enhanced local refinement, and directed exploitation in the middle and later stages. During the exploration phase, a hierarchical perturbation mechanism based on Logistic chaotic mapping and Lévy flight is introduced to enhance randomness and spatial coverage in the early search process. In the local exploitation phase, a Cauchy–Gauss hybrid mutation operator is employed to improve the algorithm’s capability to escape from local optima. In the middle and later search stages, a stochastic differential mutation strategy is incorporated to provide population-structure-based directional guidance for individuals, thereby accelerating convergence and improving optimization accuracy. Simulation results on the CEC2017 benchmark test functions indicate that CLD-RBMO demonstrates clear superiority over the original algorithm and several representative swarm intelligence optimization algorithms in terms of optimization accuracy, stability, and overall performance ranking. Convergence curve analysis confirms its dynamic performance improvements across different search stages, and the Wilcoxon rank-sum test further statistically validates the significance of the performance enhancement achieved by the proposed improvements compared with the original algorithm. Moreover, evaluations on two representative mechanical engineering optimization case studies further demonstrate the algorithm’s strong stability and engineering generalization capability.

## 1. Introduction

The concept of swarm intelligence was first proposed by Beni to describe the collective intelligent behavior emerging from a large number of simple individuals interacting locally without centralized control [1]. The essence of this paradigm lies not in the complex decision-making capability of individual agents, but in the mechanisms of information dissemination, cooperative behavior, and feedback structures within the group, which give rise to emergent effects whereby the whole exceeds the sum of its parts.

Based on this paradigm, researchers introduced principles of collective behavior into numerical optimization, which gradually evolved into swarm intelligence optimization algorithms. Such algorithms primarily simulate the collective behaviors observed in biological populations in nature, such as ant colonies, bird flocks, and bee swarms [2]. These bio-inspired algorithms achieve effective optimization of complex problems in high-dimensional search spaces through mechanisms of cooperation and information sharing [3]. Zhihua Cui et al. have suggested that Particle Swarm Optimization (PSO) and Ant Colony Optimization (ACO) are the two most representative algorithms in the field of swarm intelligence [4]. PSO simulates the cooperative behaviors of fish schools or bird flocks and updates particle positions by integrating individual historical best and global best information, thereby progressively approaching the optimal solution [5]. In contrast, ACO relies on pheromone-based indirect communication among individuals and employs positive feedback to reinforce high-quality path selection, demonstrating strong performance in path planning and combinatorial optimization problems [6].

Given their ability to remain effective in complex optimization problems where traditional mathematical or analytical methods fail, as well as their scalability and robustness [7,8]. Swarm intelligence-based optimization algorithms have been widely applied in various engineering fields, including cloud resource scheduling, optimization in the petroleum industry, multi-UAV cooperative scheduling, and pattern mining [9,10]. Meanwhile, Nandini Nayar et al. suggested that future research will increasingly focus on computational challenges in high-dimensional and data-stream environments, as well as context-aware feature selection and related research directions [11].

The Red-billed blue magpie optimizer (RBMO), proposed by Shengwei Fu et al., is a swarm intelligence optimization algorithm inspired by the behavioral mechanisms of red-billed blue magpies in natural environments, namely search for food, attacking prey, and food storage [12]. Relevant studies indicate that RBMO features a simple structure, a limited number of control parameters, and ease of implementation and application, while also demonstrating competitive performance in terms of search efficiency and convergence speed [13,14].

Following its introduction, RBMO has been increasingly applied to a wide range of practical optimization and intelligent modeling problems. For example, in energy and power systems, it has been employed for modeling and parameter identification of various electrochemical energy systems, including state-of-charge estimation of lithium-ion batteries and parameter extraction and performance characterization of proton exchange membrane fuel cells [15,16]. In computer vision, RBMO has been utilized for image processing tasks such as multi-threshold image segmentation. In medical data analysis, it has also been applied to high-dimensional discrete search tasks, including feature selection [17,18]. These applications are typically characterized by nonlinearity, high parameter dimensionality, and complex high-dimensional search spaces, demonstrating that RBMO possesses strong adaptability and can achieve effective optimization performance across diverse engineering and data-driven domains.

Although RBMO has demonstrated notable advantages in various optimization problems, previous studies have reported several limitations in complex optimization settings [19]. On the one hand, as the search iterations proceed, RBMO tends to rely heavily on existing high-quality solutions, leading to reduced population diversity and thereby limiting its global exploration capability [20]. On the other hand, insufficient coordination between global exploration and local exploitation during the search process makes RBMO prone to premature convergence in complex multimodal problems, and its convergence efficiency in the mid-to-late stages remains to be improved [21,22].

### Research Gap and Main Contributions

In recent years, to address issues in RBMO such as reduced population diversity, susceptibility to premature convergence, and insufficient convergence efficiency in the mid-to-late stages when dealing with complex optimization problems, existing studies have introduced strategies such as chaotic mapping, Lévy flight, and hybrid mutation to improve the algorithm. These methods have, to some extent, enhanced the algorithm’s exploration capability in complex search spaces and its ability to escape local optima, achieving favorable performance in certain benchmark tests.

However, a comprehensive review of existing improvement methods reveals several remaining limitations. First, most studies are still based on single-strategy enhancement or direct multi-strategy superposition, lacking clear functional differentiation and coordinated design among different mechanisms, which makes it difficult to form a stable and effective optimization structure. Second, existing methods generally do not model the search process from a stage-wise perspective, and the coordination between global exploration and local exploitation often relies on empirical parameters or random mechanisms, lacking a structured regulation framework oriented to different search stages. Third, current research mainly focuses on overall performance improvement, with limited systematic analysis of the individual contributions of each strategy and insufficient investigation of parameter sensitivity, which weakens the interpretability of algorithm design and the reliability of results to some extent.

To address these issues, this study constructs a stage-oriented mechanism-coordinated optimization framework based on the evolutionary characteristics of the search process, in which different optimization strategies are assigned to different stages according to their functional roles, thereby achieving structured coordination between exploration and exploitation. The main contributions of this work are summarized as follows:

(1) A stage-oriented mechanism coordination framework is proposed, in which the optimization process is divided into early exploration, middle adjustment, and late exploitation stages, enabling structured allocation of different search strategies.

(2) Within this framework, an early-stage exploration enhancement mechanism based on Logistic chaotic mapping and Lévy flight is designed to improve population distribution diversity and global search capability through directional perturbation and step-size diversification.

(3) A middle-stage local optimization mechanism based on Cauchy–Gauss hybrid mutation is developed, which improves the ability to escape local optima by combining large-scale jumps with fine-grained perturbations.

(4) A late-stage directed exploitation mechanism based on random differential mutation is designed to enhance convergence efficiency and stability in the mid-to-late stages of the search process.

(5) A comprehensive experimental evaluation framework is constructed, including benchmark function tests, statistical significance analysis, ablation experiments, and parameter sensitivity analysis, where key parameters are determined via Bayesian optimization to improve the systematicity of parameter selection and the reliability of results. Based on the above design, a multi-strategy improved Red-Billed Blue Magpie Optimizer, termed CLD-RBMO, is proposed [23]. The algorithm aims to maintain strong global exploration capability while improving convergence accuracy and search efficiency in complex optimization problems [24].

Finally, CLD-RBMO is compared with RBMO, PSO, WOA, MFO, DBO, SSA, SCA, HHO, and HBA on the CEC2017 benchmark test suite through optimization performance evaluation and convergence curve analysis, and the effectiveness of the proposed improvements is statistically validated using the Wilcoxon rank-sum test [25,26]. In addition, the Welded Beam Design problem and the 10-Bar Truss Optimization with Frequency Constraints problem are selected for application validation to further assess the algorithm’s applicability in practical engineering scenarios [27,28].

## 2. Red-Billed Blue Magpie Optimizer

The Red-billed Blue Magpie Optimizer (RBMO) originates from the observation and abstraction of the predatory behaviors of red-billed blue magpies [29]. Red-billed blue magpies exhibit highly flexible predatory capabilities and pronounced social behavior, typically foraging cooperatively in small groups of 2–5 individuals or larger groups exceeding 10 individuals in natural environments. In addition, red-billed blue magpies store food in tree cavities or crevices to cope with environmental fluctuations [30,31].

These behaviors establish the mathematical modeling foundation for the algorithm’s exploration and exploitation mechanisms, whereby candidate solutions are iteratively updated through three sequential phases: search for food, attacking prey, and food storage, until the termination condition is satisfied [32].

### 2.1. Search for Food

Red-billed blue magpies often explore food sources cooperatively in small groups (2–5 individuals) or large groups (>10 individuals), and this process is abstracted into two search models:

(1) Small-group cooperative search

(1) $$X_{i}(t+1)=X_{i}(t)+\left(\frac{1}{p}\sum_{m=1}^{p}X_{m}(t)-X_{rs}(t)\right)\times Rand_{1}$$

where *p* ∈ [2,5] denotes the size of the small group, randomly selected from the population. $X_{m}(t)$ represents the position of a randomly selected group member. $X_{rs}(t)$ denotes an arbitrary randomly chosen individual in the current iteration and $Rand_{1}$ is a random number uniformly distributed in the interval (0,1) [33].

(2) Large-group aggregation search

(2) $$X_{i}(t+1)=X_{i}(t)+\left(\frac{1}{q}\sum_{m=1}^{q}X_{m}(t)-X_{rs}(t)\right)\times Rand_{2}$$

where q>10 denotes the size of the large group. The meanings of the other symbols are consistent with those in the small-group model.

### 2.2. Attacking Prey

When prey is discovered, red-billed blue magpies rapidly aggregate and attempt to attack the prey [34].

Small-group attack:

(3) $$X_{i}(t+1)=X^{\mathrm{food}}(t)+CF\left(\frac{1}{p}\sum_{m=1}^{p}X_{m}(t)-X_{i}(t)\right)\times Randn_{1}$$

Large-group attack:

(4) $$X_{i}(t+1)=X^{\mathrm{food}}(t)+CF\left(\frac{1}{q}\sum_{m=1}^{q}X_{m}(t)-X_{i}(t)\right)\times Randn_{2}$$

(5) CF=1−t/T−(2⋅t/T)

where $X^{\mathrm{food}}(t)$ represents the global optimal position. Randn denotes a standard normally distributed random number. CF is a gradually decreasing convergence factor. This behavior drives individuals to converge toward the global optimal position, thereby enhancing local exploitation efficiency.

### 2.3. Food Storage

Red-billed blue magpies store surplus food in secure locations for future use.

(6) $$X_{i}(t+1)=\left\{\begin{array}{l} X_{i}(t),\;{\mathrm{if}\;\mathrm{fitness}}_{i}^{\mathrm{old}}>{\mathrm{fitness}}_{i}^{\mathrm{new}}\\ X_{i}(t+1),\;\mathrm{else} \end{array}\right.$$

This behavior preserves high-quality solutions, thereby enhancing the stability and memory capability of the algorithm [35].

## 3. Multi-Strategy Improved Red-Billed Blue Magpie Optimizer

To address the insufficient balance between exploration and exploitation in RBMO, particularly the limited exploitation capability and low convergence efficiency during the middle and late search stages, this study introduces improvements from three aspects: (1) constructing a hierarchical global perturbation mechanism based on Logistic chaos and Lévy flight. (2) Incorporating a Cauchy–Gauss hybrid mutation operator to enhance local refinement. (3) Designing a directed exploitation strategy based on random differential mutation to improve convergence efficiency in the middle and later stages. Ultimately, a multi-strategy improved Red-billed Blue Magpie Optimizer, termed CLD-RBMO, is developed.

### 3.1. Exploration Phase Enhancement Based on Logistic Chaos and Lévy Flight

In swarm intelligence-based optimization algorithms, the quality of global exploration during the early search stage directly determines the algorithm’s ability to escape local optima [36]. In the exploration phase of RBMO, two complementary global perturbation mechanisms, namely a Logistic-map-based chaotic perturbation and Lévy flight, are introduced to operate on two distinct aspects: search direction and step size [37]. Accordingly, the exploration enhancement in CLD-RBMO is decomposed into two coordinated components: directional perturbation and step-size diversification.

At the spatial level, the mechanisms operate on both the search direction and the step-size aspects. At the temporal level, chaotic perturbation decays via quadratic annealing, whereas the Lévy step size decreases through linear annealing, thereby jointly enabling strong and diverse perturbations in the early stage and weak perturbations with stable convergence in the mid-to-late stages. This stage-oriented design ensures that exploration is dominant in the early phase while gradually transitioning toward exploitation as the iteration proceeds.

#### 3.1.1. Directional Perturbation Enhancement Based on Logistic Chaos

Chaos is a class of complex behavior existing in nonlinear dynamical systems. Although its evolution is governed by deterministic equations, the system behavior exhibits pronounced pseudo-random characteristics. Specifically, minute perturbations in the initial state of the system may be rapidly amplified during the iterative process, leading to significant divergence in subsequent trajectories [38].

Owing to these properties, chaotic sequences maintain intrinsic regularity while simultaneously exhibiting strong irregularity and unpredictability. The generated sequences possess favorable uniform distribution and global ergodicity, which help expand the search range of the solution space, enable initial solutions to more adequately cover potential optimal regions, and effectively alleviate premature convergence of the algorithm [39]. Therefore, in this study, Logistic chaos is introduced to perturb the search direction in the early stage, rather than directly replacing the original RBMO update rule.

Chaotic mappings commonly employed in optimization algorithms include the Logistic map, Henon map, and Lorenz map, among others. Among these, the Logistic map is widely adopted due to its simple mathematical form, low computational cost, and significant chaotic properties, and its definition is given as follows:

(7) $$x_{t+1}=\mu x_{t}\left(1-x_{t}\right),\;\mu \in (0,4],\;x_{t}\in (0,1)$$

where $x_{t}$ denotes the chaotic variable at iteration t, $x_{t+1}$ denotes the updated chaotic variable, and μ is the control parameter of the Logistic map. This equation is used to generate a chaotic sequence for directional perturbation.

Unlike conventional approaches that directly superimpose chaotic noise at every iteration, this study adopts a periodic activation strategy, updating the chaotic sequence only when

(8) $$t\;\mathrm{mod}\;k_{\mathrm{freq}}=0$$

where t is the current iteration number and $k_{\mathrm{freq}}$ denotes the activation interval of chaotic perturbation. This condition is introduced to prevent excessive disturbance from being imposed at every iteration and to maintain rhythmic directional correction during the search process.

This mechanism prevents excessive interference of chaos with the primary search process and ensures rhythmically controlled perturbations throughout the iterative procedure.

Furthermore, to introduce directional characteristics into the perturbation rather than confining it to non-negative chaotic values, the generated chaotic sequence is further shifted and linearly rescaled to the symmetric interval (−1,1) as follows:

(9) $$c_{t}=2\left(x_{t}-0.5\right)\in (-1,1)$$

where $c_{t}$ denotes the rescaled chaotic variable. This transformation allows the perturbation to act in both positive and negative directions, thereby avoiding one-sided directional bias.

Since chaotic perturbation should exert a strong influence in the early stage and gradually weaken in the later stage to facilitate convergence, a chaotic directional perturbation coefficient based on a quadratic annealing function is introduced:

(10) $$\begin{array}{l} \Delta_{\mathrm{chaos}}(t)=c_{t}\cdot \eta_{t}\\ \eta_{t}={\left(1-\frac{t}{T}\right)}^{2} \end{array}$$

where $\Delta_{\mathrm{chaos}}(t)$ denotes the chaotic directional perturbation coefficient, $\eta_{t}$ denotes the annealing factor controlling perturbation intensity, and T denotes the maximum number of iterations. This formulation ensures that the directional perturbation is strong in the early stage and gradually weakens as the search progresses.

After obtaining the perturbation coefficient, the next step is to embed it into the original RBMO update structure so that chaos acts as a directional correction rather than an independent position jump. It should be noted that the chaotic perturbation is not directly added to the position variable. Instead, it functions as a directional correction term within the original step-size update equation, enhancing the ability to escape local optima by modulating the magnitude of movement toward the population mean:

(11) $$X_{i}^{t+1}=X_{i}^{t}+S_{i}+\Delta_{\mathrm{chaos}}(t)\cdot \left(m_{i}-X_{i}^{t}\right)$$

where $X_{i}^{t}$ and $X_{i}^{t+1}$ denote the positions of the i-th individual at iterations t and t+1, respectively, and $m_{i}$ denotes the mean-guided search direction. This equation mainly controls the directional perturbation of individuals and is used in the early stage to enhance global exploration capability, where $m_{i}\in \left\{X_{p}^{\mathrm{mean}},X_{q}^{\mathrm{mean}}\right\}$ denotes the mean individual and $S_{i}$ represents the step size of the original RBMO algorithm:

(12) $$S_{i}=\left\{\begin{array}{c} \left(X_{p}^{\mathrm{mean}}-X_{R1}\right)\cdot u,\;\mathrm{if}\;r<\varepsilon \\ \left(X_{q}^{\mathrm{mean}}-X_{R1}\right)\cdot u,\;\mathrm{otherwise} \end{array}\right.$$

where $X_{p}^{\mathrm{mean}}$ and $X_{q}^{\mathrm{mean}}$ denote the mean positions of the randomly selected small group and large group, respectively. $X_{R1}$ denotes a randomly selected individual in the current population. u∼U(0,1) is a uniformly distributed random number controlling the step magnitude. r∼U(0,1) is the probability-switching random number and ε=0.5 is the probability threshold. Compared with the original RBMO, the introduced Logistic chaotic perturbation does not alter the basic search framework, but strengthens directional diversity and reduces the risk of premature convergence. In the following subsection, Lévy flight is introduced to complement this mechanism from the step-size perspective, thereby forming a direction-step coordinated exploration strategy.

#### 3.1.2. Step-Jump Enhancement Based on Lévy Flight

Complementing the directional perturbation introduced by Logistic chaos, Lévy flight is employed to enhance the search process from the perspective of step-size diversification. Lévy flight is a special type of random walk characterized by a heavy-tailed step-length distribution, such that the random walk includes not only numerous short-distance moves but also, with a certain probability, a few extremely long jumps [40]. This alternating pattern of short and long movements enables rapid cross-regional global exploration in the early search stage while maintaining moderate perturbations in later iterations, thereby effectively avoiding entrapment in local optima.

Common Lévy flight approaches include the Mantegna method and Lévy walk, among others [41]. In this study, the classical Mantegna method is employed to generate Lévy-distributed steps in a dimension-wise manner. The step vector of the i-th individual in a d-dimensional search space is defined as

(13) $$L_{i}=L_{i1},L_{i2},\ldots ,L_{id}$$

where $L_{i}$ denotes the Lévy step vector of the i-th individual, and d is the dimensionality of the search space.

For each dimension j=1,…,d,

(14) $$L_{ij}=\frac{u_{ij}\sigma_{u}}{{\left|v_{ij}\right|}^{1/\beta }}$$

where

(15) $$\begin{array}{c} u_{ij}∼N(0,1),\;v_{ij}∼N(0,1),\\ \sigma_{u}={\left[\frac{\Gamma (1+\beta )\sin(\pi \beta /2)}{\Gamma \left(\frac{1+\beta }{2}\right)\beta 2^{(\beta -1)/2}}\right]}^{1/\beta } \end{array}$$

where $u_{ij}$ and $v_{ij}$ are independent standard Gaussian random variables, Γ(⋅) denotes the Gamma function, and β is the shape parameter of the Lévy distribution, typically set in the interval (1,2]. This formulation generates Lévy-distributed step lengths with heavy-tailed characteristics.

In classical Lévy flight, the sign of each step component is entirely determined by $u_{ij}$, implying that the search direction is solely governed by Gaussian noise, which may introduce directional bias. To mitigate this issue, an additional random sign term is incorporated, and the step vector is redefined as follows:

(16) $$\begin{array}{c} {\tilde{L}}_{i}=\left({\tilde{L}}_{i1},\ldots ,{\tilde{L}}_{id}\right),\;{\tilde{L}}_{ij}=sign\left(r_{ij}-0.5\right)L_{ij}\\ r_{ij}∼U(0,1) \end{array}$$

where $r_{ij}$ is a uniformly distributed random variable used to assign equal probability to positive and negative directions, thereby improving directional symmetry of the perturbation.

Consequently, in each dimension, positive or negative Lévy step lengths are selected with equal probability, thereby ensuring better statistical symmetry of perturbations in both directions.

The intensity of Lévy flight should vary across different iteration stages; therefore, a linear annealing factor is introduced into the step length:

(17) $$\lambda (t)=\lambda_{0}\left(1-\frac{t}{T}\right),\;t=1,\ldots ,T$$

where λ(t) denotes the adaptive scaling factor of the Lévy step, $\lambda_{0}$ is the initial step coefficient, t is the current iteration number, and T is the maximum number of iterations. This formulation ensures that larger step sizes are used in the early stage, while smaller steps are adopted in later stages to improve convergence stability.

Its decay rate is slower than the quadratic annealing decay of the Logistic chaotic term, enabling Lévy flight to continue dominating long-range jump behavior after chaotic perturbations gradually diminish.

To maintain a balance between exploration and exploitation, Lévy flight is not applied to all individuals but only to a subset of the population. Lévy flight is applied only to a subset of individuals in the population, while the remaining individuals are updated according to the original algorithm. For individuals participating in Lévy flight, the position update is defined as

(18) $$X_{i}^{t+1}=X_{i}^{t}+\lambda (t)\left({\tilde{L}}_{i}⊗r_{i}\right)$$

where $X_{i}^{t}$ and $X_{i}^{t+1}$ denote the positions of the i-th individual at iterations t and t+1, respectively. ⊗ denotes element-wise multiplication, $r_{i}∼U{\left(0,1\right)}^{d}$ is a random vector controlling the perturbation magnitude in each dimension. This equation primarily controls the step size of individuals and is used in the early stage to enhance global exploration capability through long-range jumps.

Due to the stochastic nature of Lévy flight, the generated jump does not necessarily yield a better solution than the current position. To prevent perturbations from degrading high-quality individuals, the original food storage mechanism is applied following the Lévy update. Let the Lévy-updated position be denoted as $X_{i}^{\mathrm{new}}$. The final position is then determined according to the following criterion:

(19) $$X_{i}^{t+1}=\left\{\begin{array}{l} X_{i}^{\mathrm{new}},\mathrm{if}\;f\left(X_{i}^{\mathrm{new}}\right)<f\left(X_{i}^{t}\right)\\ X_{i}^{t},\mathrm{otherwise} \end{array}\right.$$

where f(⋅) denotes the objective (fitness) function. This mechanism ensures that Lévy-based perturbations do not deteriorate solution quality while preserving beneficial exploratory moves.

### 3.2. Local Refinement Strategy Based on Cauchy–Gauss Mutation

After the exploration phase, the algorithm gradually enters the middle stage, where improving local search capability and escaping from local optima become critical. In the later stages of iteration, individuals in the population are prone to becoming trapped in local optimal regions, resulting in weakened exploitation capability. Studies indicate that during the convergence phase, priority updating should be applied to key individuals, and introducing a hybrid distribution mutation mechanism such as Cauchy–Gauss mutation can enhance search flexibility and solution diversity [42]. The candidate solution generated by the hybrid mutation is defined as

(20) $$X_{i}^{mut}(t)=X_{i}^{t}\left(1+c\left[\beta_{1}(t)C_{i}+\beta_{2}(t)G_{i}\right]\right)$$

where $X_{i}^{t}\in {ℜ}^{d}$ denotes the position of the individual at i-th iteration t, and $X_{i}^{mut}(t)$ denotes the mutated candidate solution. The function $\tau (t)={\left(\frac{t}{T}\right)}^{\beta_{\exp}}$ is a dynamic weighting factor, where T denotes the maximum number of iterations and $\beta_{\exp}$ controls the rate of transition, with $\beta_{1}(t)=1-\tau (t)$ and $\beta_{2}(t)=\tau (t)$. The vector $C_{i}=\left(C_{i1},\ldots ,C_{id}\right)$ follows a Cauchy distribution $C_{i}∼Cauchy(0,1)$, representing heavy-tailed perturbations, while $G_{i}=\left(G_{i1},\ldots ,G_{id}\right)$ follows a Gaussian distribution $G_{i}∼N(0,1)$, representing fine-grained local perturbations. The parameter c is a scaling coefficient controlling the overall perturbation intensity.

The mutated individuals are still subjected to the food storage mechanism, and Equation (19) is applied to prevent perturbations from degrading existing high-quality solutions. This mutation mechanism mainly controls the local perturbation intensity and is applied in the middle stage to balance large-scale jumps and fine-grained adjustments. The Cauchy–Gauss mutation does not undertake a new global search but rather serves to prevent the algorithm from being trapped in local optima.

### 3.3. Directed Exploitation Enhancement Strategy Based on Random Differential Mutation

After the local refinement stage, the algorithm further enters the late stage, where convergence speed and stability become the primary objectives. Differential mutation is a stochastic optimization method based on population evolution mechanisms and has been widely applied to solving complex continuous optimization problems since its introduction. The algorithm constructs new candidate solutions using differential vectors among population individuals and iteratively updates them through crossover and greedy selection strategies, thereby maintaining structural simplicity while exhibiting strong global search capability [43].

Following the Cauchy–Gauss-based random local refinement, the algorithm transitions into an exploitation phase focused on convergence toward promising regions. At this stage, if directionless random perturbations are still employed, the update step may fail to effectively steer the search toward high-quality regions, thereby slowing convergence and degrading search efficiency. In contrast, the directional update step introduced by differential mutation effectively alleviates this issue. The candidate position of the i-th individual at iteration t is defined as

(21) $$V_{i}^{t}=X_{r_{1}}^{t}+F_{t}\left(X_{r_{2}}^{t}-X_{r_{3}}^{t}\right)$$

where $X_{r_{1}}^{t}$, $X_{r_{2}}^{t}$, and $X_{r_{3}}^{t}$ denote the positions of three distinct individuals randomly selected from the current population, and $V_{i}^{t}$ denotes the generated candidate solution. This formulation constructs a directional search vector to guide individuals toward promising regions.

The scaling factor is defined as

(22) $$F_{t}=F_{\min}+rand\left(F_{\max}-F_{\min}\right)$$

where $F_{t}$ is the dynamic scaling factor controlling the step size of the differential mutation, $F_{\max}$ and $F_{\min}$ denote its lower and upper bounds, respectively, and  rand∼U(0,1) is a uniformly distributed random number.

Similarly, to prevent degradation of existing high-quality individuals, the update rule remains

(23) $$X_{i}^{t+1}=\left\{\begin{array}{l} V_{i}^{t},\mathrm{if}\;f\left(V_{i}^{t}\right)<f\left(X_{i}^{t}\right)\\ X_{i}^{t},\mathrm{otherwise} \end{array}\right.$$

where f(⋅) denotes the objective (fitness) function. This mechanism mainly controls the convergence direction and speed in the late stage, improving convergence efficiency and stability compared with the original RBMO.

### 3.4. Pseudocode and Flowchart of CLD-RBMO

**Figure 1:**
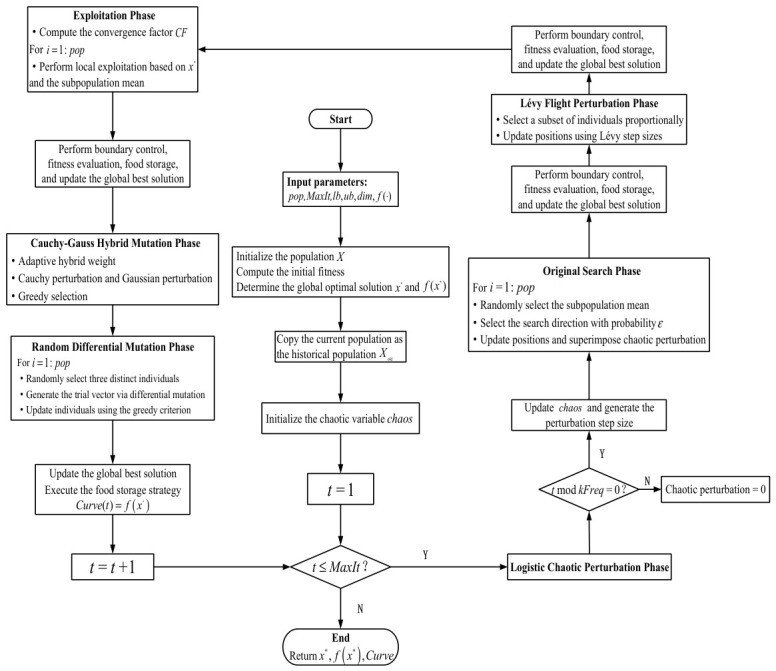
The flowchart of the CLD-RBMO algorithm.

The pseudocode of CLD-RBMO is shown in Table 1, and the flowchart is shown in Figure 1.

### 3.5. Time Complexity Analysis of CLD-RBMO

In the original RBMO algorithm, let the population size be pop , the problem dimension be dim , the maximum number of iterations be MaxIt , and the computational cost of the objective function be f(dim) . The initialization phase requires generating the initial population and evaluating its fitness, with a time complexity of O(pop⋅dim+pop⋅f(dim)) . During iteration, RBMO mainly consists of the Search and Exploitation phases, both of which require dimension-wise position updates and boundary handling for all individuals in the population, followed by fitness evaluation at the end of each phase. Therefore, the time complexity per iteration is O(pop⋅(dim+f(dim))). Ignoring constant and lower-order terms, the overall time complexity of RBMO is O(MaxIt⋅pop⋅(dim+f(dim))).

In the CLD-RBMO algorithm, the initialization phase is identical to that of RBMO, and its time complexity remains unchanged. During the iteration phase, CLD-RBMO introduces strategies such as Logistic chaotic perturbation, Lévy flight, Cauchy–Gaus hybrid mutation, and random differential mutation on top of the original search and exploitation mechanisms. Among these, chaotic perturbation involves only constant-time operations, while the other improvement strategies perform dimension-wise updates at the individual level, keeping the time complexity at a linear order. Therefore, the time complexity per iteration of the improved algorithm remains O(pop⋅(dim+f(dim))) , and the overall time complexity is O(MaxIt⋅pop⋅(dim+f(dim))).

In summary, CLD-RBMO maintains the same time complexity as the original RBMO, indicating that the improvement process does not increase the order of time complexity and demonstrating that CLD-RBMO retains good computational efficiency after incorporating multiple enhancement strategies.

## 4. Algorithm Simulation and Results Analysis

### 4.1. Simulation Environment Setup

The simulation environment in this study consists of a Windows 11 64-bit operating system with 16 GB memory, an AMD Ryzen 7 7735H @ 3.2 GHz CPU, and MATLAB R2024b as the simulation platform.

### 4.2. Comparative Algorithms and Parameter Settings

To comprehensively evaluate the optimization performance of CLD-RBMO, nine representative swarm intelligence optimization algorithms proposed in recent years are selected as benchmark algorithms, including Particle Swarm Optimization (PSO) [44], Whale Optimization Algorithm (WOA) [45], Harris Hawks Optimization (HHO) [46], Sparrow Search Algorithm (SSA) [47], Sine Cosine Algorithm (SCA) [48], Moth-Flame Optimization (MFO) [49], Dung Beetle Optimization (DBO) [50], and Honey Badger Algorithm (HBA) [51], and comparisons are conducted with the original Red-billed Blue Magpie Optimizer (RBMO). The parameter settings of the nine algorithms are listed in Table 2.

For the proposed CLD-RBMO, the parameter configuration is determined through a Bayesian optimization process conducted over a predefined parameter search space. The optimization involves key parameters associated with the three enhancement strategies, including those related to Logistic chaotic perturbation, Lévy flight, Cauchy–Gauss mutation, and differential mutation. The objective function of the Bayesian optimization is defined as the average fitness value over multiple representative benchmark functions, so that the obtained parameter configuration achieves stable and balanced performance across different types of optimization problems.

The final parameter values of CLD-RBMO are summarized in Table 2. In addition, the probability threshold ε follows the same setting as the original RBMO, ensuring that the basic search mechanism is preserved while the proposed strategies enhance the optimization capability.

To ensure fairness in comparison, all algorithms are executed under identical experimental conditions, including the same problem dimension, population size, maximum number of iterations, and number of independent runs. For the comparative algorithms, the parameter settings recommended in their original references are directly adopted without additional tuning. Therefore, the performance differences observed in the experiments can be primarily attributed to the algorithmic design rather than parameter adjustments.

### 4.3. Test Functions

The CEC2017 benchmark test functions are employed as the evaluation platform for the optimization algorithms in this study. This benchmark suite comprises representative categories of objective functions, including unimodal, multimodal, hybrid, and composition functions, which comprehensively reflect algorithm performance in terms of search efficiency, global optimization capability, and stability in complex environments. Since the second function in CEC2017 has been officially removed, the remaining 29 functions are selected for experimentation. The formulas and related information of the benchmark functions are presented in Table 3.

### 4.4. Convergence Curve Analysis of RBMO and Benchmark Algorithms

In this simulation experiment, the search dimension for all algorithms is set to 50, the population size is 50, each algorithm is independently executed 30 times under identical parameter settings, and the maximum number of iterations is 500. The detailed results are presented in Table 4.

For performance evaluation, the average best value (avg) and standard deviation (std) are employed for comprehensive assessment. The average value measures optimization accuracy, while the standard deviation reflects stability across multiple independent runs. Accordingly, the average value is adopted as the primary comparison criterion, and when averages are identical or close, the standard deviation is used as the secondary criterion for ranking algorithm performance. The ranking results are presented in Table 5.

As shown in Table 4, in terms of convergence accuracy, CLD-RBMO outperforms the original RBMO on 22 test functions and achieves the globally optimal average performance on 21 of them. In terms of stability, CLD-RBMO exhibits a smaller standard deviation than the original algorithm on 21 functions and outperforms all comparative algorithms on 17 functions, indicating strong stability and robustness.

According to Table 5, CLD-RBMO achieves leading rankings on most test functions, with an average rank of 1.38, placing first overall and demonstrating superior overall performance compared with the original RBMO. Further analysis by function type shows that for unimodal functions (F1–F3), CLD-RBMO consistently outperforms the original algorithm and achieves globally optimal rankings, exhibiting strong convergence accuracy and exploitation capability, with notable improvement in convergence efficiency. For multimodal functions (F4–F10), CLD-RBMO outperforms the original algorithm on more than half of the functions and maintains leading overall rankings, indicating that the premature convergence tendency of the original algorithm in complex multimodal problems has been effectively alleviated. For hybrid functions (F11–F20), CLD-RBMO demonstrates stable ranking performance and outperforms the original RBMO on 70% of the functions, suggesting that the exploration–exploitation balance has been enhanced. For composition functions (F21–F30), CLD-RBMO achieves superior results compared with the original algorithm on 80% of the functions, all of which are globally optimal, further demonstrating its robustness and adaptability in highly complex search spaces.

### 4.5. Convergence Curve Analysis of RBMO and Representative Algorithms

**Figure 2:**
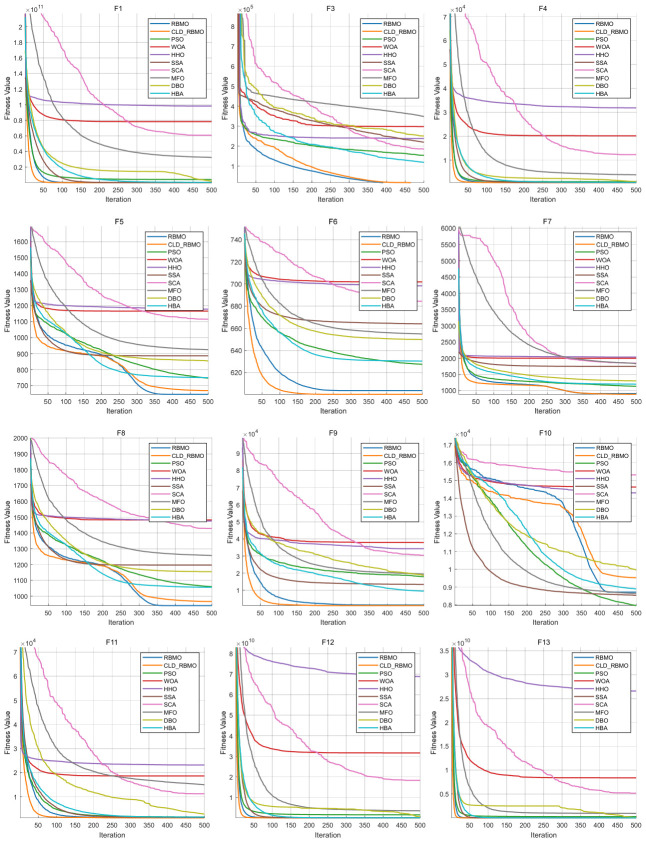

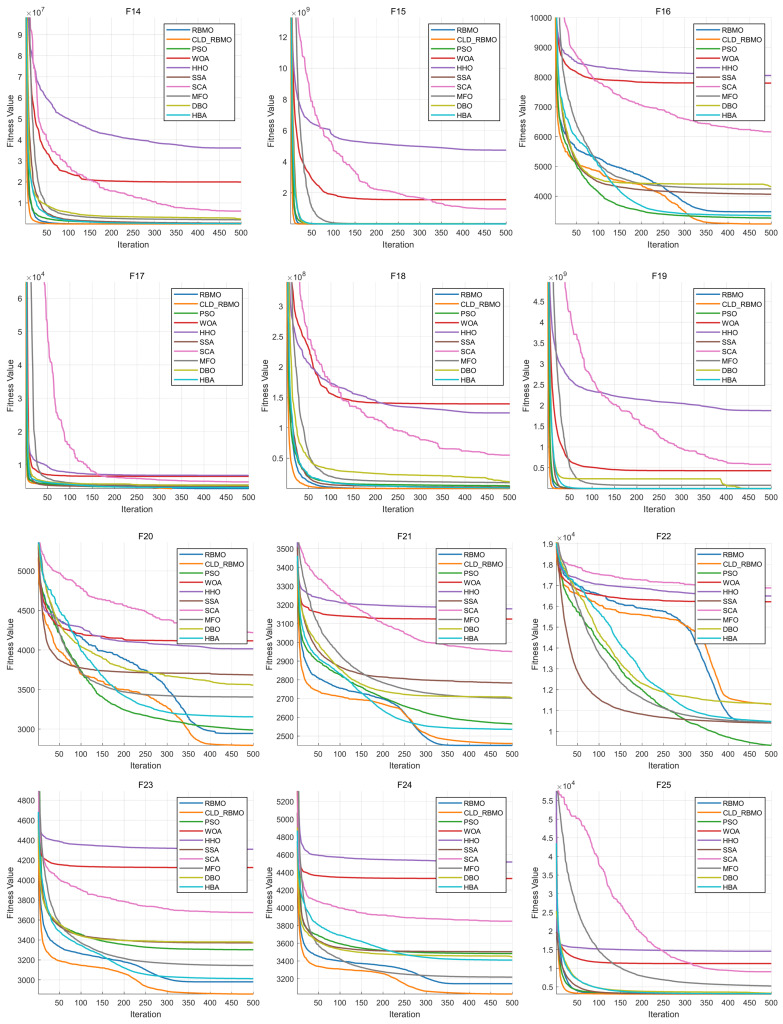

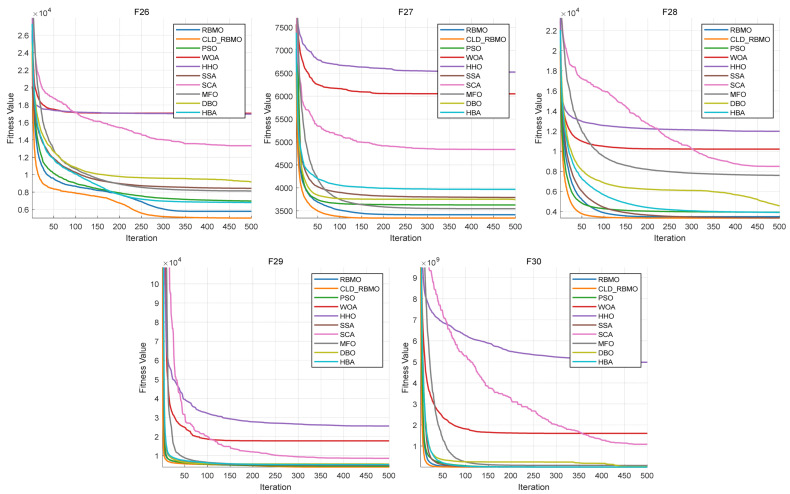
Convergence curves of CLD-RBMO and comparative algorithms (D = 50).

**Figure 3:**
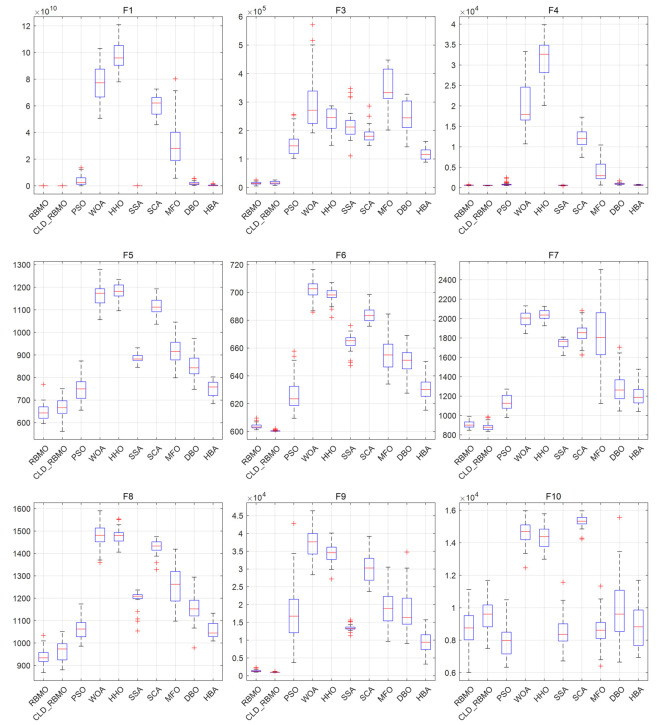

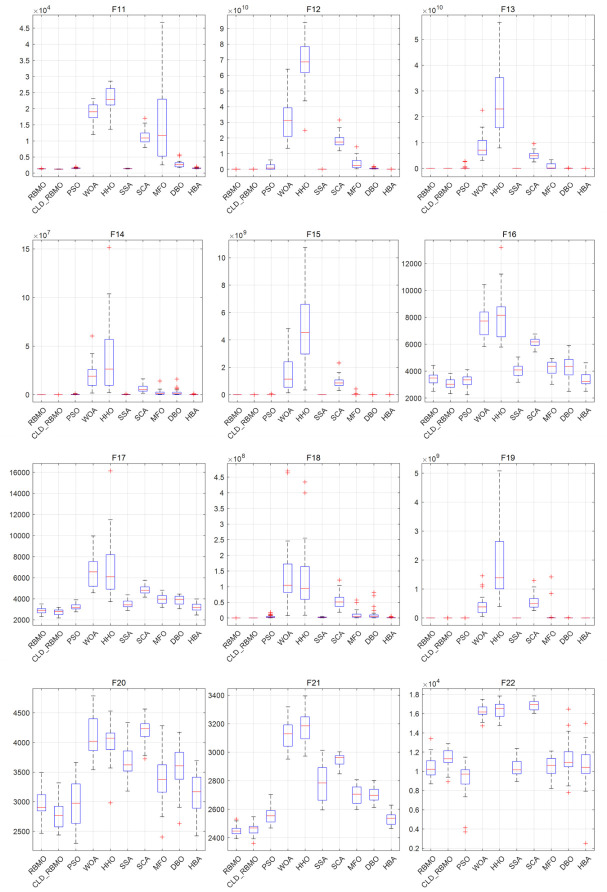

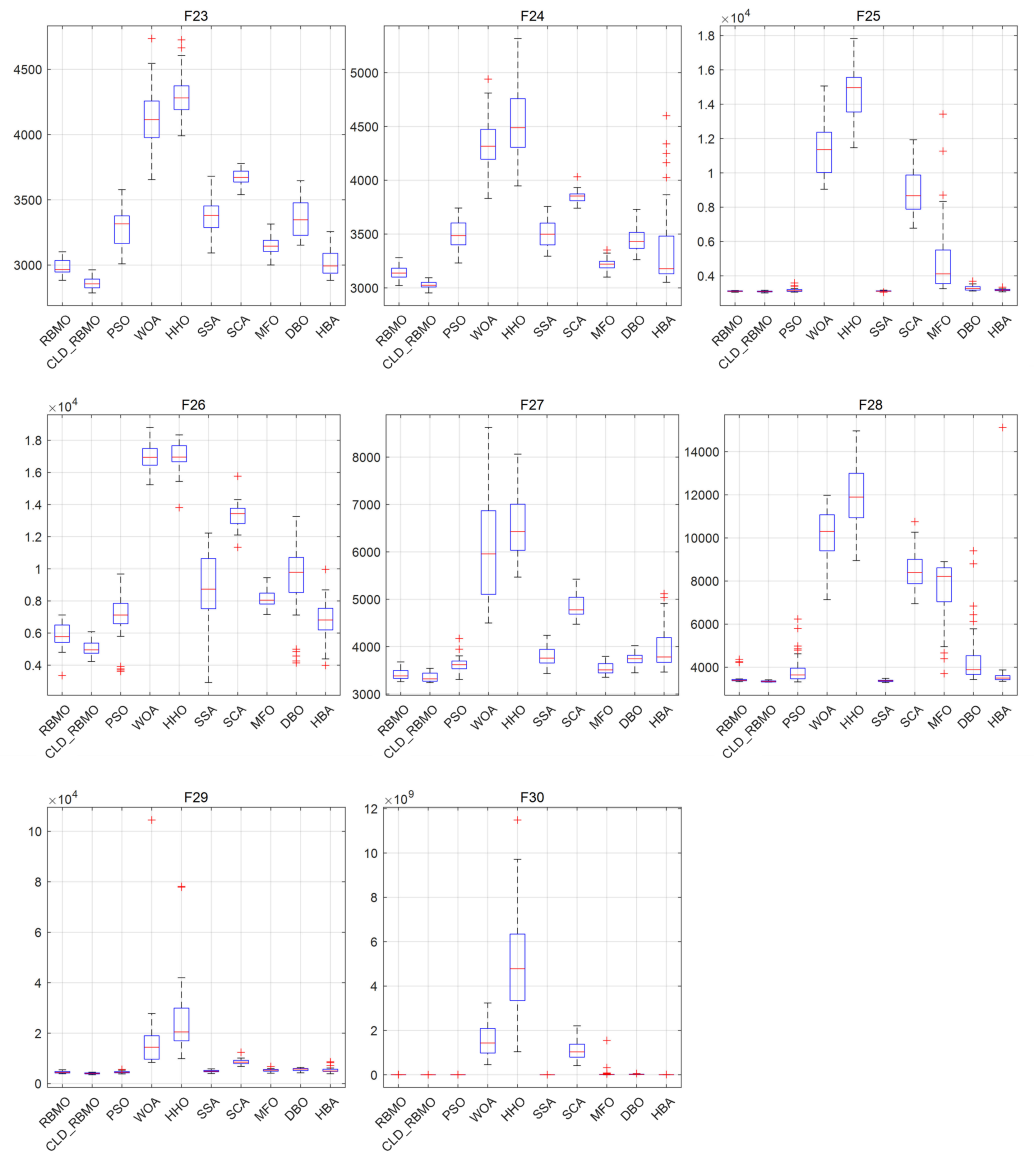
The box plots of CLD-RBMO and comparative algorithms (D = 50).

To further analyze the dynamic behavior characteristics of CLD-RBMO at different stages of the search process, convergence curves of all algorithms are comparatively analyzed using 50-dimensional test functions as examples. For each test function, all algorithms are independently executed 30 times under identical parameter settings, and representative convergence curves close to the average performance are plotted, as shown in Figure 2. Meanwhile, to reflect the distribution characteristics of different algorithms over multiple independent runs, the corresponding box plots are presented in Figure 3.

In the experiments on unimodal functions (F1–F3) and multimodal functions (F4–F10), the improved algorithm demonstrates a markedly faster convergence speed than most comparative algorithms and exhibits stronger capability to escape local optima on functions such as F1, F4, F6, F7, and F9. This indicates that the introduction of Logistic chaotic perturbation and Lévy flight mechanisms in the early stage effectively enhances global exploration capability. Moreover, the Cauchy–Gauss mutation operator provides moderate random perturbations during the search process, thereby alleviating the premature convergence tendency of the original algorithm in complex multimodal problems.

In the more challenging experiments involving hybrid functions (F11–F20) and composition functions (F21–F30), CLD-RBMO achieves the best results on most test functions (e.g., F11–F16, F18–F20, F23–F28, and F30). These results indicate that the improved algorithm achieves a better balance between global exploration and local exploitation, demonstrates strong robustness and generalization ability, and exhibits superior search capability in complex landscapes. This further confirms that the proposed multi-strategy framework operates synergistically and leads to overall performance improvements over the original RBMO.

The box plots further corroborate the convergence curves. For most test functions, the boxes are relatively compact, exhibiting low variability and few outliers. These results indicate that, compared with the original algorithm, the improved method enhances exploration capability in the early stage, improves the ability to escape local optima in the middle stage, and achieves higher convergence efficiency and accuracy in the late stage, while maintaining strong overall stability.

### 4.6. Wilcoxon Rank-Sum Test

To further verify the statistical significance of the performance differences between CLD-RBMO and the original RBMO, the Wilcoxon rank-sum test is employed on each test function to conduct nonparametric statistical analysis of the experimental results obtained from multiple independent runs. The corresponding test results are presented in Table 6.

In Table 6, the *p*-value represents the significance level obtained from the Wilcoxon rank-sum test, and when p<0.05 , it indicates that the performance difference between CLD-RBMO and the original algorithm on the current test function is statistically significant. When p≥0.05 , it indicates that the performance difference between the two algorithms on the corresponding test function is not statistically significant. “+/−” denote “significant difference/non-significant difference,” respectively.

The results in Table 6 indicate that CLD-RBMO differs significantly from the original algorithm on 20 functions, with most *p*-values far below 0.05. Therefore, compared with the original algorithm, CLD-RBMO not only achieves better performance but also exhibits statistically significant differences.

### 4.7. Ablation Study

In the ablation study, the search dimension for all variants is set to 30, the population size is 30, each variant is independently executed 20 times under identical parameter settings, and the maximum number of iterations is 500. The detailed results are presented in Table 7. To further analyze the contribution of each component in CLD-RBMO and verify the effectiveness of the proposed multi-strategy coordinated framework, an ablation study is conducted by removing one mechanism at a time while keeping the remaining components unchanged.

Specifically, three ablation variants are constructed: LD-RBMO, CD-RBMO, and CL-RBMO. LD-RBMO removes the Cauchy–Gauss mutation mechanism, thereby eliminating the local refinement capability in the middle stage while retaining the exploration mechanism (Logistic chaos and Lévy flight) and the directed exploitation mechanism (differential mutation). CD-RBMO removes the early-stage exploration enhancement mechanism, including both Logistic chaotic perturbation and Lévy flight, while preserving the Cauchy–Gauss mutation and differential mutation mechanisms. CL-RBMO removes the differential mutation mechanism, thus weakening the directed exploitation capability in the later stage while maintaining the exploration and local refinement mechanisms.

Through this design, the individual contribution of each mechanism can be quantitatively evaluated, and the necessity of their coordinated interaction across different search stages can be systematically verified.

For performance evaluation, the average best value (avg) and standard deviation (std) are employed for comprehensive assessment. The average value measures optimization accuracy, while the standard deviation reflects stability across multiple independent runs. Accordingly, the average value is adopted as the primary comparison criterion, and when averages are identical or close, the standard deviation is used as the secondary criterion for ranking the performance of different ablation variants.

The ranking results are presented in Table 8.

As shown in Table 7, in terms of convergence accuracy, CLD-RBMO outperforms the ablation variants on 12 benchmark functions and achieves competitive performance on most of the remaining functions. Specifically, CLD-RBMO attains the best average results on F1, F3, F7, F11, F13, F14, F19, F24, F26, F29, and F30, demonstrating its strong overall optimization capability. In contrast, LD-RBMO, CD-RBMO, and CL-RBMO exhibit varying degrees of performance degradation after removing different components, indicating that each mechanism contributes positively to the algorithm. In terms of stability, CLD-RBMO achieves relatively smaller standard deviations on multiple functions such as F1, F3, F7, and F13, indicating improved robustness. However, in certain functions such as F12 and F18, some ablation variants (e.g., CL-RBMO or CD-RBMO) show competitive or even better stability, suggesting that individual mechanisms may have stronger effects under specific problem characteristics. According to Table 8, CLD-RBMO achieves the best overall performance with a mean rank of 1.86, ranking first among all ablation variants. LD-RBMO, CD-RBMO, and CL-RBMO obtain mean ranks of 2.52, 2.72, and 2.90, respectively, confirming that removing any component leads to a decline in overall performance.

Further analysis reveals that different mechanisms contribute at different stages. When the exploration mechanism (Logistic chaos and Lévy flight) is removed (CD-RBMO), the algorithm shows degraded performance on several multimodal functions such as F4, F8, and F9, indicating reduced global exploration capability. When the local refinement mechanism (Cauchy–Gauss mutation) is removed (LD-RBMO), the performance decreases on functions such as F15 and F16, suggesting weaker ability to escape local optima. When the directed exploitation mechanism (differential mutation) is removed (CL-RBMO), the algorithm exhibits poorer convergence accuracy on functions such as F10, F18, and F22, indicating slower convergence in the later stage.

Overall, the results demonstrate that the three mechanisms in CLD-RBMO play complementary roles in different stages of the optimization process, and their coordinated integration is essential for achieving stable and high-quality optimization performance.

### 4.8. Sensitivity Analysis of Key Parameters

To further investigate the robustness of the proposed CLD-RBMO algorithm, a sensitivity analysis is conducted on several key parameters. The objective is to examine how variations in these parameters influence the optimization performance under different problem characteristics. Considering the diversity and computational cost of the CEC2017 benchmark suite, a representative subset of six functions (F1, F3, F6, F9, F23, and F26) is selected. These functions cover unimodal, multimodal, and composite landscapes, enabling the evaluation of parameter influence under different search scenarios while maintaining computational efficiency. All experiments in this section are carried out under identical settings, where the problem dimension is 30, the population size is 30, the maximum number of iterations is 500, and each experiment is independently repeated 20 times. During the sensitivity analysis, only the target parameter is varied, while all other parameters remain fixed.

For clarity, the parameters are grouped according to the stages in which they primarily take effect, including the early exploration stage, the middle local refinement stage, and the late directed exploitation stage. Specifically, the early exploration stage involves the Logistic parameter α, the chaos frequency $k_{\mathrm{freq}}$, and the Lévy distribution parameter β. The middle local refinement stage mainly includes the scaling coefficient, and the late directed exploitation stage is characterized by the differential scaling factor (represented by $F_{\max}$ while fixing $F_{\min}$).

#### 4.8.1. Early Exploration Stage

In the early exploration stage, the algorithm mainly relies on the Logistic chaos mechanism and Lévy flight strategy to enhance population diversity and expand the search range. The parameters in this stage directly affect the perturbation intensity and the distribution of search trajectories.

**Figure 4:**
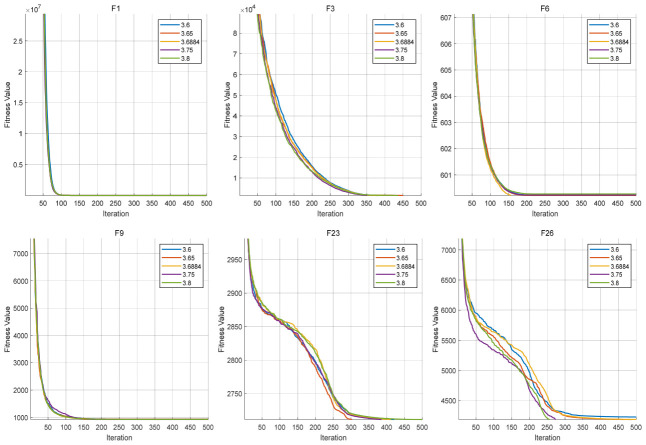
Convergence curves under different values of the Logistic parameter α.

The Logistic parameter α is first analyzed. It controls the dynamic behavior of the chaotic sequence and thus determines the strength of early-stage exploration. In this experiment, several representative values of α are tested, while all other parameters remain fixed. The convergence behaviors are shown in Figure 4, and the corresponding average fitness values are listed in Table 9.

As illustrated in Figure 4, for F1, F3, F6, and F9, the convergence curves under different values of α are highly similar, with only minor deviations observed. This indicates that the influence of α is limited for these functions, and the algorithm maintains stable performance within the tested parameter range. In contrast, for F23 and F26, more noticeable differences can be observed. Especially in F26, the convergence curves show clear separation in the middle and later stages, suggesting that different values of α lead to different search trajectories. This implies that in complex composite landscapes, the Logistic parameter has a more direct impact on guiding the search process.

Overall, the results indicate that the algorithm exhibits low sensitivity to α on relatively simple functions, while showing moderate sensitivity on complex functions. Nevertheless, no significant performance degradation is observed across the tested range, indicating that the selected parameter setting provides stable behavior.

**Figure 5:**
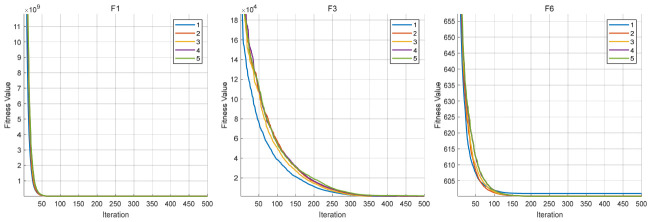

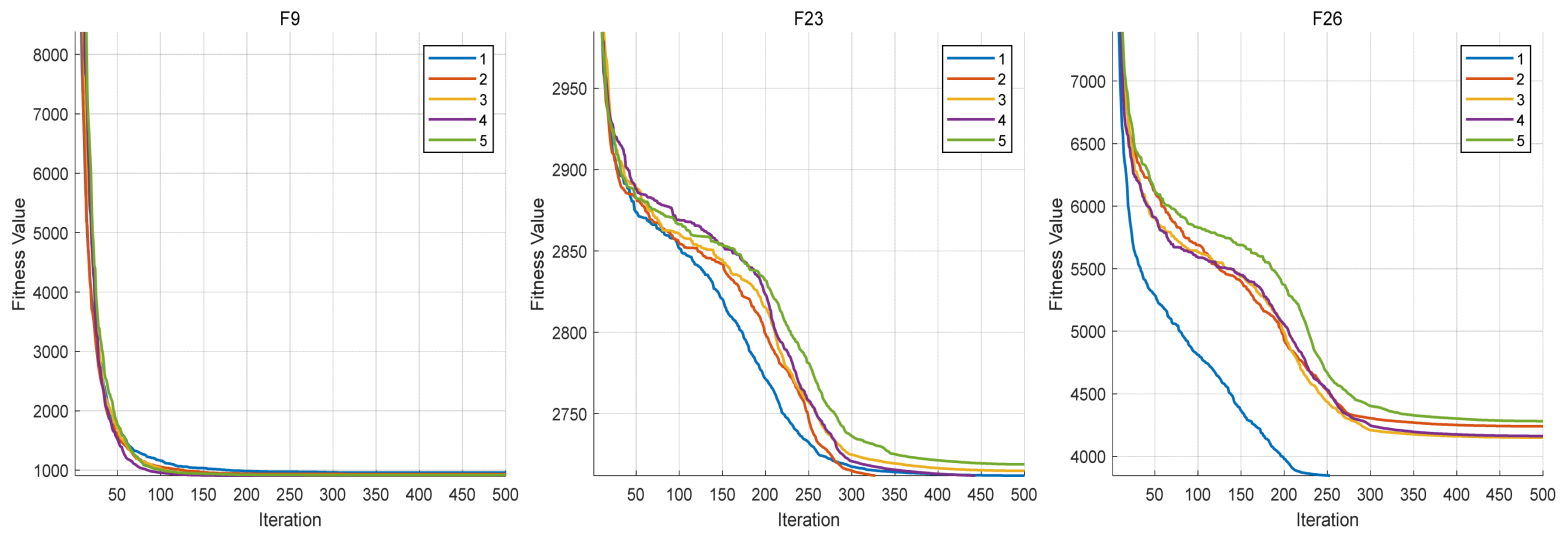
Convergence curves under different values of the chaos frequency $k_{\mathrm{freq}}$.

The chaos frequency $k_{\mathrm{freq}}$ is further analyzed. This parameter determines the activation frequency of the Logistic chaotic perturbation and therefore affects how often the directional correction mechanism participates in the search process. In this experiment, several representative values of $k_{\mathrm{freq}}$ are tested, while all other parameters remain fixed. The convergence behaviors are shown in Figure 5, and the corresponding average fitness values are listed in Table 10.

As illustrated in Figure 5, for F1, F3, F6, and F9, the convergence curves under different values of $k_{\mathrm{freq}}$ remain relatively close, indicating that the algorithm is not highly sensitive to the chaos frequency on these functions. Although slight differences can still be observed in the intermediate convergence process, the final fitness values remain within a narrow range, suggesting that the exploration mechanism maintains stable effectiveness under different activation frequencies. For F23 and F26, the influence of $k_{\mathrm{freq}}$ becomes more noticeable. In particular, on F26, the convergence trajectories under different values of $k_{\mathrm{freq}}$ show clearer separation in the middle and later stages, indicating that the activation frequency of chaotic perturbation affects the subsequent search path in complex composite landscapes. Compared with simple functions, the effect of $k_{\mathrm{freq}}$ on the search process is therefore more direct in these complex scenarios.

Overall, the results indicate that the algorithm does not exhibit strong sensitivity to the chaos frequency $k_{\mathrm{freq}}$ on relatively simple functions, whereas on complex composite functions, different values of $k_{\mathrm{freq}}$ lead to observable differences in convergence behavior. Nevertheless, the overall performance remains within a relatively stable range across the tested interval, indicating that the selected parameter setting provides good robustness rather than relying on a single isolated value. In the early exploration stage, the algorithm further incorporates the Lévy flight mechanism to introduce long-range perturbations, which enhances the ability to escape local optima and improves global search coverage. The Lévy distribution parameter β plays a key role in controlling the step-length distribution of the random walk, thereby directly affecting the balance between local and global movements.

**Figure 6:**
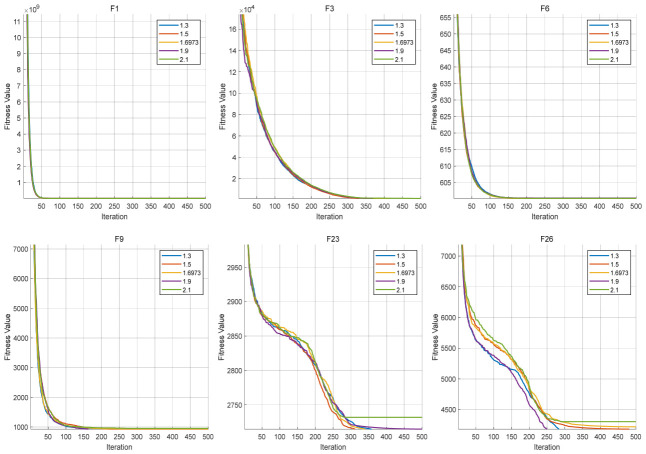
Convergence curves under different values of the Lévy distribution parameter β.

The Lévy distribution parameter β is then analyzed. It determines the heaviness of the tail in the Lévy distribution and thus influences the probability of large-step exploration. In this experiment, several representative values of β are tested, while all other parameters remain fixed. The convergence behaviors are shown in Figure 6, and the corresponding average fitness values are listed in Table 11.

From the results on unimodal functions (F1 and F3), the convergence curves under different β values are relatively close, indicating that the algorithm is not highly sensitive to β in simple landscapes. This suggests that the exploitation capability is mainly governed by subsequent refinement and exploitation mechanisms rather than the Lévy-based exploration component. For multimodal functions (F6 and F9), the convergence curves under different β values remain largely consistent, with only minor fluctuations observed in limited intervals. This indicates that, within the tested range, the influence of β on the overall search behavior is relatively weak. Although β theoretically affects the step-length distribution of Lévy flights, its effect is moderated by the coordinated interaction of multiple mechanisms, resulting in negligible differences in convergence performance. For complex hybrid and composition functions (F23 and F26), the influence of β becomes more noticeable. Distinct separation among convergence curves can be observed, particularly in the middle stage of the optimization process. This implies that the Lévy flight parameter plays a more critical role in navigating complex landscapes, where both global exploration and local adaptation are required.

Overall, within the tested range, the algorithm exhibits stable performance across different β values without significant degradation. Meanwhile, the observed variations on complex functions indicate that β provides an effective control over the exploration scale, allowing the algorithm to adapt to different landscape complexities while maintaining robustness.

#### 4.8.2. Middle Local Refinement Stage

In the middle local refinement stage, the algorithm employs the Cauchy–Gauss hybrid mutation strategy to enhance local search capability and improve solution accuracy. The scaling coefficient c plays a key role in this stage, as it controls the amplitude of perturbations and thus directly affects the balance between exploration and exploitation during the refinement process.

**Figure 7:**
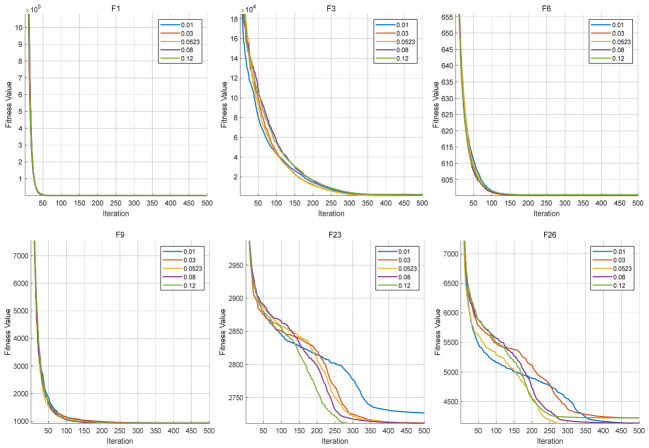
Convergence curves under different values of scaling coefficient c.

The scaling coefficient c is analyzed in this section. It determines the intensity of the hybrid mutation and influences how aggressively candidate solutions are updated around the current search region. In this experiment, several representative values of c are tested, while all other parameters remain fixed. The convergence behaviors are shown in Figure 7, and the corresponding average fitness values are listed in Table 12.

From the results on unimodal functions (F1 and F3), the convergence curves under different values of c are highly similar, with the curves on F1 almost completely overlapping, while a relatively clearer separation can be observed on F3. This indicates that the algorithm is not particularly sensitive to the scaling coefficient in most simple landscapes, but certain functions (such as F3) exhibit higher dependence on the perturbation magnitude, leading to observable differences in convergence behavior. For multimodal functions (F6 and F9), the convergence curves under different values of c remain largely consistent, showing only minor variations throughout the iterations. This suggests that, in these cases, the influence of the scaling coefficient on the search trajectory is limited, and the overall optimization process is mainly governed by the algorithm’s inherent search dynamics rather than the perturbation intensity. For complex hybrid and composition functions (F23 and F26), the influence of c becomes more evident. The convergence curves exhibit clearer separation under different parameter settings, particularly in the middle and later stages. This indicates that the scaling coefficient plays a critical role in controlling the refinement dynamics in complex landscapes, where an appropriate perturbation magnitude is necessary to balance convergence speed and solution quality.

Overall, within the tested range, the algorithm maintains stable performance across different values of c without significant degradation. Meanwhile, the observed variations across different functions indicate that the effect of c is not uniform, but depends on the landscape characteristics of the problem. In particular, while the influence of c is limited in most cases, it becomes more noticeable in functions such as F3 and in complex composition problems, where the local refinement process is more sensitive to perturbation magnitude. This suggests that the scaling coefficient primarily acts as a fine-tuning factor in the refinement stage, rather than a dominant driver of the overall search behavior.

#### 4.8.3. Late Directed Exploitation Stage

In the late directed exploitation stage, the algorithm mainly relies on the random differential mutation strategy to improve convergence efficiency and enhance solution accuracy. In this stage, the upper bound of the scaling factor $F_{\max}$ plays an important role, since it determines the maximum intensity of the differential perturbation and thus directly affects the convergence behavior in the later search process.

**Figure 8:**
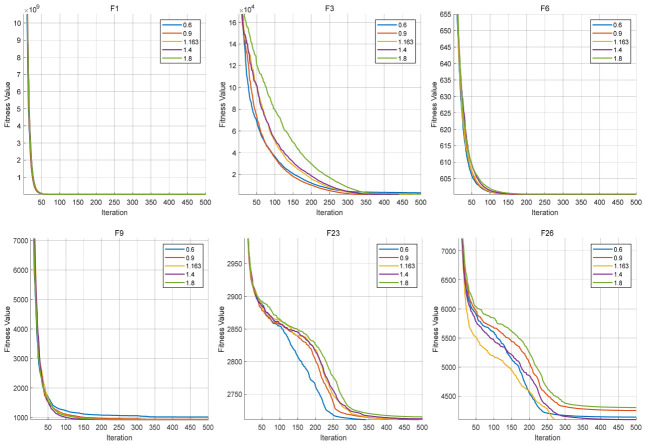
Convergence curves under different values of upper bound of scaling factor $F_{\max}$.

The upper bound of the scaling factor $F_{\max}$ is analyzed in this section. It controls the upper range of the differential mutation coefficient and influences the search step size during the directed exploitation process. In this experiment, several representative values of $F_{\max}$ are tested, while all other parameters remain fixed. The convergence behaviors are shown in Figure 8, and the corresponding average fitness values are listed in Table 13.

From the results on unimodal functions (F1 and F3), the convergence curves under different values of $F_{\max}$ remain relatively close, with the curves on F1 almost completely overlapping, while slightly more noticeable deviations can be observed on F3, indicating that the algorithm is not highly sensitive to this parameter in simple landscapes. This suggests that when the search space is relatively smooth, different upper bounds of the scaling factor lead to only limited changes in the final convergence behavior. For multimodal functions (F6 and F9), the convergence curves under different values of $F_{\max}$ remain relatively close, showing only minor differences throughout the iterations. This indicates that the influence of $F_{\max}$ on the search trajectory is still limited in these landscapes, and the directed exploitation process remains stable under different parameter settings.

For complex composition functions (F23 and F26), the influence of $F_{\max}$ is more evident. The convergence curves under different parameter settings show clearer separation, particularly in the later stage. This indicates that the upper bound of the scaling factor has a more direct effect on the search trajectory in complex landscapes, where the trade-off between convergence speed and exploitation stability becomes more critical.

Overall, within the tested range, the algorithm maintains relatively stable performance under different values of $F_{\max}$, without significant performance degradation. At the same time, the observed differences across functions indicate that the influence of $F_{\max}$ increases with landscape complexity, being minimal on simple functions (F1), moderate on multimodal functions (F6 and F9), and more pronounced on functions such as F3, F23, and F26. This suggests that $F_{\max}$ mainly serves as a regulator of exploitation intensity in complex scenarios, rather than a dominant factor affecting the overall convergence behavior.

### 4.9. Mechanism Interpretation of the Performance Improvement

The superior performance of CLD-RBMO is not attributed to a single strategy, but rather to the coordinated interaction of multiple mechanisms across different search stages. Unlike the original RBMO, which mainly relies on the inherent transition between exploration and exploitation behaviors, CLD-RBMO introduces explicit stage-oriented regulation, enabling different search strategies to dominate at appropriate phases of the optimization process. In the early stage, the integration of Logistic chaotic mapping and Lévy flight significantly enhances population diversity and expands the exploration range. This improvement effectively delays premature convergence and increases the probability of discovering promising regions in complex landscapes. The superiority observed on multimodal functions such as F4, F6, F8, and F10 suggests that the enhanced global exploration capability plays a crucial role in handling multiple local optima. In the middle stage, the Cauchy–Gauss hybrid mutation provides a balance between exploration and exploitation by combining long-distance jumps with fine-grained local perturbations. This mechanism allows the algorithm to maintain adaptability after the initial exploration phase and improves its ability to escape local optima. The competitive performance on functions such as F11, F14, and F18 indicates that this hybrid mutation is particularly effective for problems with irregular landscapes and complex basin structures. In the late stage, the random differential mutation strategy introduces directionally guided updates based on population information, which enhances convergence efficiency and solution accuracy. This mechanism is especially beneficial for unimodal functions, where precise exploitation is required. The superior results on F1, F3, F20, F26, and F30 suggest that the directed exploitation capability significantly accelerates convergence toward the global optimum.

It is also observed that CLD-RBMO does not consistently achieve the best performance on every single function. This indicates that while the multi-strategy framework improves overall robustness and average performance, the additional perturbation mechanisms may not always provide advantages for functions with relatively simple landscapes or highly concentrated optimal regions. Therefore, the main strength of CLD-RBMO lies in its balanced performance and adaptability across diverse problem types, rather than dominance in all individual cases.

Furthermore, the ablation study demonstrates that removing any individual component leads to a noticeable performance decline, confirming that the proposed strategies are complementary rather than redundant. The sensitivity analysis shows that CLD-RBMO maintains stable performance within a reasonable parameter range, indicating that its effectiveness does not rely on precise parameter tuning. These findings collectively suggest that the performance improvement of CLD-RBMO is supported by both mechanism-level synergy and parameter-level robustness.

## 5. Application Verification in Engineering Design Problems

The performance of an algorithm is reflected not only in its optimization capability on benchmark test functions but also in its effectiveness in practical engineering applications. To further validate the applicability and effectiveness of the proposed CLD-RBMO in engineering optimization scenarios, it is applied to the welded beam design problem and the 10-bar truss design problem in the field of mechanical design, and compared with nine representative swarm intelligence optimization algorithms. In the experiments, the population size of each algorithm is set to 30, the maximum number of function evaluations is 300, and each algorithm is independently executed 30 times under identical parameter settings to ensure fairness and reliability of the results.

### 5.1. Welded Beam Design Problem

The welded beam design problem is a classical continuous single-objective constrained optimization problem in structural optimization, widely used to evaluate the optimization capability of intelligent algorithms under complex engineering constraints. This problem aims to minimize the fabrication cost of a welded beam structure while satisfying multiple mechanical constraints, including shear stress, normal stress, and buckling load, featuring strong variable coupling and high nonlinearity, thereby comprehensively reflecting algorithm performance in practical engineering design problems.

The welded beam design model adopted in this study is derived from the engineering optimization test problems proposed by Kumar et al. [34,35]. The problem consists of 4 continuous design variables and 5 inequality constraints, and its mathematical model can be formulated as follows:

(1) Design variables:

(24) $$x=\left[x_{1},x_{2},x_{3},x_{4}\right]=[h,l,t,b]$$

where h,l,t, and b denote the weld thickness, weld length, beam height, and beam thickness, respectively.

(2) Objective function:

(25) $$\min f(x)=0.04811tb(l+14)+1.10471h^{2}l$$

which represents minimizing the total fabrication cost of the welded beam subject to all constraints.

(3) Constraints:

(26) $$\left\{\begin{array}{l} g_{1}(x):h-b\leq 0,\\ g_{2}(x):\delta (x)-\delta_{\max}\leq 0,\\ g_{3}(x):P\leq P_{c}(x),\\ g_{4}(x):\tau (x)-\tau_{\max}\leq 0,\\ g_{5}(x):\sigma (x)-\sigma_{\max}\leq 0. \end{array}\right.$$

which correspond to the weld-to-beam thickness relationship constraint, maximum deflection constraint, buckling load constraint, maximum shear stress constraint, and maximum normal stress constraint, respectively.

(4) Variable bounds:

(27) $$\begin{array}{c} 0.125\leq h\leq 2\\ 0.1\leq l\leq 10\\ 0.1\leq t\leq 10\\ 0.1\leq b\leq 2 \end{array}$$

**Figure 9:**
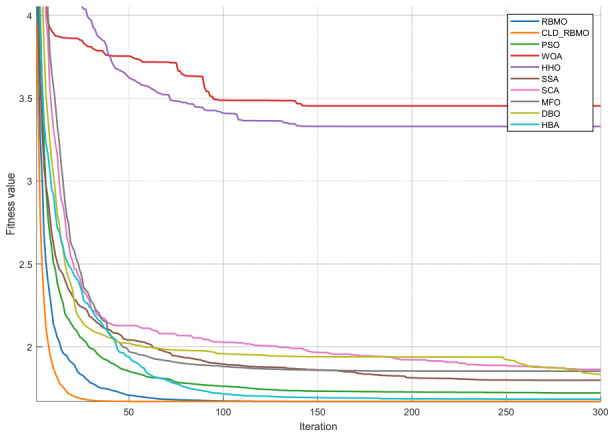
The convergence curve for the Welded Beam Design problem.

The convergence curve of the experiment is shown in Figure 9, and the final results are presented in Table 14. CLD-RBMO achieves the minimum cost of 1.6702177263E+00 for the welded beam design problem.

### 5.2. Ten-Bar Truss Optimization with Frequency Constraints Problem

The 10-bar truss design problem is a classical continuous constrained optimization problem in structural optimization, commonly used to evaluate the comprehensive optimization capability of algorithms under structural lightweight design and dynamic performance constraints. This problem aims to minimize material consumption while satisfying frequency performance requirements, featuring clear engineering significance and research value.

The 10-bar truss design model adopted in this study is also derived from the engineering optimization test problems proposed by Kumar et al. [34,36]. The problem consists of 10 continuous design variables and 3 inequality constraints, and its mathematical formulation can be expressed as follows:

(1) Design variables:

(28) $$x=\left[x_{1},x_{2},\ldots ,x_{10}\right]=\left[A_{1},A_{2},\ldots ,A_{10}\right]$$

where $A_{i}$ denotes the cross-sectional area of the i-th member, and i=1,2,…,10.

(2) Objective function:

(29) $$\min f(x)=\sum_{i=1}^{10}L_{i}\rho A_{i}$$

which minimizes the total weight of the 10-bar truss subject to frequency constraints.

(3) Constraints:

(30) $$\left\{\begin{array}{c} g_{1}(x):\frac{7}{\omega_{1}(x)}-1\leq 0,\\ g_{2}(x):\frac{15}{\omega_{2}(x)}-1\leq 0,\\ g_{3}(x):\frac{20}{\omega_{3}(x)}-1\leq 0. \end{array}\right.$$

which represent the natural frequency constraints of the 10-bar truss.

(4) Variable bounds:

The bounds of the cross-sectional areas of the members are given as

(31) $$6.45\times 10^{-5}\leq A_{i}\leq 5\times 10^{-3},\;i=1,2,\ldots ,10.$$

**Figure 10:**
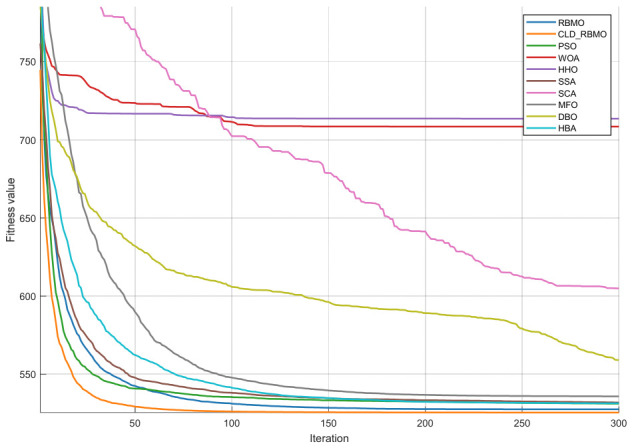
The convergence curve for the 10-bar truss design problem.

The convergence curve of the experiment is shown in Figure 10, and the results are presented in Table 15. CLD-RBMO achieves the minimum weight of 5.244547E+02 in the 10-bar truss design problem.

## 6. Conclusions, Limitations, and Future Work

This study proposed a multi-strategy improved Red-Billed Blue Magpie Optimizer, termed CLD-RBMO, to address the decline of population diversity, the insufficient balance between exploration and exploitation, and the low convergence efficiency of the original RBMO in complex optimization problems. The proposed method integrates three coordinated mechanisms, namely Logistic-chaos- and Lévy-flight-based early exploration enhancement, Cauchy–Gauss hybrid mutation for middle-stage local refinement, and random differential mutation for late-stage directed exploitation, thereby constructing a stage-oriented optimization framework.

From the perspective of algorithm design, the proposed improvements enhance the search process in a structured manner. In the early stage, Logistic chaotic perturbation and Lévy flight improve search-direction diversity and step-size diversity, which helps enlarge the search region and delay premature convergence. In the middle stage, the Cauchy–Gauss hybrid mutation improves the capability of escaping local optima by combining occasional large jumps with fine-grained perturbations. In the late stage, the random differential mutation introduces directionally guided updates based on population structure information, which improves convergence efficiency and final solution accuracy. The experimental results demonstrate that CLD-RBMO achieves clear performance improvements over the original RBMO and most comparative algorithms. On the CEC2017 benchmark suite, the proposed algorithm shows better optimization accuracy, stronger stability, and superior overall ranking performance. The convergence curves further verify that CLD-RBMO exhibits faster and more effective search behavior across different stages, while the Wilcoxon rank-sum test confirms that the improvement over the original RBMO is statistically significant on most test functions. In addition, the ablation study demonstrates that the three proposed mechanisms play complementary roles in the optimization process, and the sensitivity analysis indicates that the algorithm maintains relatively stable performance within a reasonable parameter range. These results together support the effectiveness, robustness, and interpretability of the proposed multi-strategy framework.

Future research may be carried out in several directions. First, the proposed framework can be extended to more complex optimization scenarios, such as large-scale optimization, multi-objective optimization, dynamic optimization, and discrete or combinatorial optimization problems. Second, adaptive or self-tuning parameter control strategies can be introduced to further improve the flexibility and portability of the algorithm across different tasks. Third, more real-world engineering applications and cross-domain validation experiments can be considered to further assess the generalization capability and practical value of CLD-RBMO. In addition, future work may further simplify the coordination mechanism or develop lightweight variants to improve implementation efficiency while preserving optimization performance.

## Figures and Tables

**Table 1:** The pseudocode of CLD-RBMO algorithm.

**Algorithm 1.** Pseudocode of CLD-RBMO	
**Input:** Population size pop, maximum number of iterations MaxIt, Lower bound lb, upper bound ub, Problem dimension dim, objective function f(⋅)	
**Output:** Optimal solution $x^{*},$ optimal fitness value $f\left(x^{*}\right),$ convergence curve Curve	
(1).	Initialize the population X compute the initial fitness, and determine the global optimal solution $x^{*}$ and $f\left(x^{*}\right)$
(2).	Copy the current population as the historical population $X_{\mathrm{old}}$
(3).	Initialize the chaotic variable chaos
(4).	For t=1:MaxIt
(5).	**%%Logistic chaotic perturbation phase**
(6).	If t mod kFreq=0, update the chaotic variable and generate the perturbation step size
(7).	Otherwise, set the chaotic perturbation to 0
(8).	**%%Original search phase**
(9).	For i=1:pop
(10).	Randomly select subgroup means $X_{p}$ and $X_{q}$
(11).	Select the search direction according to probability ε
(12).	Update the individual position and superimpose chaotic perturbation
(13).	End For
(14).	Perform boundary control, fitness evaluation, and food storage, and update the global optimal solution
(15).	**%%Lévy Flight perturbation phase**
(16).	Select a portion of individuals according to a specified ratio
(17).	Generate Lévy step lengths and update their positions
(18).	Perform boundary control, fitness evaluation, and food storage, and update the global optimal solution
(19).	**%%Exploitation phase**
(20).	Compute the convergence factor CF
(21).	For i=1:pop
(22).	Perform local exploitation based on the global best and subgroup means
(23).	End For
(24).	Apply boundary control, fitness evaluation, and food storage, and update the global best
(25).	**%%Cauchy–Gauss hybrid mutation phase**
(26).	Compute hybrid weights according to iteration progress
(27).	Generate Cauchy and Gaussian perturbations
(28).	Construct mutated individuals and perform greedy selection
(29).	**%%Random differential mutation phase**
(30).	For each individual i
(31).	Randomly select three distinct individuals
(32).	Perform differential mutation to generate a trial vector
(33).	Update individuals using a greedy criterion
(34).	End For
(35).	Update the global best
(36).	Apply the food storage strategy
(37).	Record the best value of the current iteration in Curve(t)
(38).	End For
(39).	Return $x^{*}$, $f\left(x^{*}\right),$ and Curve

**Table 2:** The parameter settings of the algorithms.

Algorithm	Algorithm Parameters	Values
RBMO	Random threshold ε	0.5
PSO	Control parameter a	Linearly decreased from 2 to 0
WOA	Velocity upper bound $V_{\max}$	30
	Inertia weight ω	1
	Cognitive learning factor $c_{1}$	1.5
	Social learning factor $c_{2}$	1.5
MFO	Control parameter a	Linearly decreased from −1 to −2
	Spiral constant b	1
DBO	Deflection coefficient k	0.1
SSA	Discoverer ratio P	0.2
	Discoverer update threshold r	0.8
SCA	Control parameter a	Linearly decreased from 2 to 0
	Switching probability r	0.5
HHO	Escaping energy coefficient $E_{1}$	Linearly decreased from 2 to 0
	Initial energy $E_{0}$	Randomly generated within [−1,1]
HBA	Intensity adjustment factor β	6
	Attenuation coefficient C	2
CLD-RBMO	Probability threshold ε	0.5
	Logistic parameter α	3.6884
	Chaos frequency $k_{\mathrm{freq}}$	2
	Lévy distribution parameter β	1.6973
	Base step coefficient $\lambda_{0}$	0.0825
	Step ratio coefficient r	0.0895
	Scaling coefficient c	0.0523
	Exponent parameter $\beta_{\exp}$	3.0355
	Lower bound of scaling factor $F_{\min}$	0.3993
	Upper bound of scaling factor $F_{\max}$	1.1630

**Table 3:** CEC2017 benchmark test functions.

No.	Function Name	Dimension	Optimal Value
F1	Shifted and Rotated Bent Cigar Function	${\left[-100,100\right]}^{D}$	100
F3	Shifted and Rotated Zakharov Function	${\left[-100,100\right]}^{D}$	300
F4	Shifted and Rotated Rosenbrock’s Function	${\left[-100,100\right]}^{D}$	400
F5	Shifted and Rotated Rastrigin’s Function	${\left[-100,100\right]}^{D}$	500
F6	Shifted and Rotated Expanded Scaffer’s F7 Function	${\left[-100,100\right]}^{D}$	600
F7	Shifted and Rotated Lunacek Bi_Rastrigin Function	${\left[-100,100\right]}^{D}$	700
F8	Shifted and Rotated Non-Continuous Rastrigin’s Function	${\left[-100,100\right]}^{D}$	800
F9	Shifted and Rotated Levy Function	${\left[-100,100\right]}^{D}$	900
F10	Shifted and Rotated Schwefel’s Function	${\left[-100,100\right]}^{D}$	1000
F11	Hybrid Function 1	${\left[-100,100\right]}^{D}$	1100
F12	Hybrid Function 2	${\left[-100,100\right]}^{D}$	1200
F13	Hybrid Function 3	${\left[-100,100\right]}^{D}$	1300
F14	Hybrid Function 4	${\left[-100,100\right]}^{D}$	1400
F15	Hybrid Function 5	${\left[-100,100\right]}^{D}$	1500
F16	Hybrid Function 6	${\left[-100,100\right]}^{D}$	1600
F17	Hybrid Function 7	${\left[-100,100\right]}^{D}$	1700
F18	Hybrid Function 8	${\left[-100,100\right]}^{D}$	1800
F19	Hybrid Function 9	${\left[-100,100\right]}^{D}$	1900
F20	Hybrid Function 10	${\left[-100,100\right]}^{D}$	2000
F21	Composition Function 1	${\left[-100,100\right]}^{D}$	2100
F22	Composition Function 2	${\left[-100,100\right]}^{D}$	2200
F23	Composition Function 3	${\left[-100,100\right]}^{D}$	2300
F24	Composition Function 4	${\left[-100,100\right]}^{D}$	2400
F25	Composition Function 5	${\left[-100,100\right]}^{D}$	2500
F26	Composition Function 6	${\left[-100,100\right]}^{D}$	2600
F27	Composition Function 7	${\left[-100,100\right]}^{D}$	2700
F28	Composition Function 8	${\left[-100,100\right]}^{D}$	2800
F29	Composition Function 9	${\left[-100,100\right]}^{D}$	2900
F30	Composition Function 10	${\left[-100,100\right]}^{D}$	3000

**Table 4:** Results of benchmark test functions (D = 50).

Function		RBMO	CLD-RBMO	GWO	WOA	HHO	SSA	SCA	MFO	DBO	HBA
F1	min	2.56E+05	4.59E+02	6.86E+08	3.50E+10	3.84E+10	4.40E+04	1.67E+10	3.66E+08	2.82E+06	4.13E+06
	std	6.92E+05	3.25E+03	2.19E+09	7.94E+09	8.15E+09	1.17E+05	3.90E+09	9.12E+09	4.81E+08	1.67E+08
	avg	1.11E+06	4.67E+03	4.60E+09	4.30E+10	5.51E+10	1.47E+05	2.35E+10	1.09E+10	5.90E+08	9.05E+07
F3	min	3.42E+03	1.77E+03	7.69E+04	1.10E+05	8.07E+04	4.04E+04	5.46E+04	1.27E+05	4.59E+04	3.72E+04
	std	3.23E+03	2.65E+03	1.85E+04	6.28E+04	3.79E+03	9.06E+03	2.01E+04	7.24E+04	2.19E+04	9.72E+03
	avg	8.91E+03	5.40E+03	9.67E+04	2.75E+05	9.20E+04	5.50E+04	1.01E+05	2.17E+05	8.96E+04	5.78E+04
F4	min	4.90E+02	4.37E+02	5.63E+02	3.70E+03	8.67E+03	4.70E+02	2.23E+03	5.21E+02	5.16E+02	4.96E+02
	std	3.48E+01	3.20E+01	1.39E+02	3.06E+03	3.07E+03	2.32E+01	1.56E+03	6.39E+02	1.02E+02	3.61E+01
	avg	5.29E+02	5.05E+02	7.26E+02	1.05E+04	1.43E+04	5.06E+02	4.22E+03	9.47E+02	6.18E+02	5.43E+02
F5	min	5.52E+02	5.35E+02	6.41E+02	8.59E+02	8.04E+02	6.08E+02	8.12E+02	6.17E+02	6.33E+02	5.77E+02
	std	2.10E+01	2.13E+01	2.52E+01	3.12E+01	4.71E+01	6.98E+01	2.49E+01	4.35E+01	4.82E+01	2.81E+01
	avg	5.85E+02	5.90E+02	6.90E+02	9.31E+02	9.16E+02	7.50E+02	8.43E+02	7.07E+02	7.06E+02	6.30E+02
F6	min	6.02E+02	6.00E+02	6.06E+02	6.67E+02	6.77E+02	6.27E+02	6.59E+02	6.25E+02	6.27E+02	6.14E+02
	std	2.31E+00	4.60E-01	5.07E+00	1.04E+01	8.13E+00	1.31E+01	5.96E+00	1.03E+01	1.17E+01	7.14E+00
	avg	6.05E+02	6.01E+02	6.16E+02	6.87E+02	6.90E+02	6.52E+02	6.69E+02	6.38E+02	6.42E+02	6.25E+02
F7	min	7.83E+02	7.77E+02	8.37E+02	1.30E+03	1.32E+03	1.02E+03	1.16E+03	9.12E+02	9.28E+02	8.73E+02
	std	2.93E+01	2.43E+01	4.29E+01	6.95E+01	4.78E+01	9.58E+01	4.91E+01	1.40E+02	8.56E+01	6.45E+01
	avg	8.33E+02	8.18E+02	9.24E+02	1.45E+03	1.46E+03	1.24E+03	1.24E+03	1.13E+03	1.05E+03	9.71E+02
F8	min	8.45E+02	8.53E+02	9.09E+02	1.09E+03	1.08E+03	9.44E+02	1.06E+03	9.45E+02	9.08E+02	8.69E+02
	std	2.41E+01	2.38E+01	2.44E+01	2.97E+01	2.95E+01	2.14E+01	2.41E+01	4.68E+01	3.36E+01	3.10E+01
	avg	8.85E+02	8.94E+02	9.52E+02	1.13E+03	1.15E+03	9.84E+02	1.11E+03	1.02E+03	9.57E+02	9.19E+02
F9	min	9.79E+02	9.08E+02	1.40E+03	8.70E+03	7.98E+03	4.44E+03	6.24E+03	4.46E+03	4.78E+03	1.42E+03
	std	1.97E+02	6.31E+01	8.25E+02	1.84E+03	1.97E+03	4.07E+02	2.34E+03	1.91E+03	1.54E+03	1.43E+03
	avg	1.15E+03	9.38E+02	2.32E+03	1.15E+04	1.16E+04	5.27E+03	9.56E+03	7.27E+03	8.02E+03	4.02E+03
F10	min	4.06E+03	4.82E+03	4.68E+03	8.04E+03	8.13E+03	4.26E+03	7.65E+03	4.27E+03	4.71E+03	4.77E+03
	std	7.20E+02	5.96E+02	5.98E+02	4.30E+02	5.20E+02	7.83E+02	4.19E+02	7.01E+02	1.26E+03	1.25E+03
	avg	5.43E+03	5.78E+03	5.77E+03	8.76E+03	8.78E+03	5.57E+03	8.78E+03	5.66E+03	7.21E+03	6.35E+03
F11	min	1.20E+03	1.13E+03	2.50E+03	8.20E+03	7.82E+03	1.16E+03	2.38E+03	1.78E+03	1.42E+03	1.24E+03
	std	3.65E+01	3.06E+01	1.76E+03	4.81E+03	2.84E+03	6.02E+01	1.02E+03	5.45E+03	7.76E+02	8.06E+01
	avg	1.25E+03	1.17E+03	6.12E+03	1.49E+04	1.22E+04	1.30E+03	4.35E+03	6.81E+03	2.20E+03	1.37E+03
F12	min	3.35E+04	2.78E+04	1.31E+07	3.45E+09	3.11E+09	1.12E+05	1.61E+09	3.16E+06	2.78E+06	2.61E+05
	std	8.06E+05	6.83E+05	1.91E+08	3.80E+09	3.82E+09	1.75E+06	8.73E+08	2.21E+08	8.23E+07	3.21E+06
	avg	6.69E+05	6.59E+05	2.99E+08	7.80E+09	1.09E+10	2.39E+06	2.99E+09	1.69E+08	8.55E+07	4.26E+06
F13	min	4.25E+03	1.40E+03	7.64E+04	1.43E+08	5.73E+08	3.80E+03	3.18E+08	1.39E+04	2.85E+04	8.92E+03
	std	2.02E+04	2.15E+04	2.35E+08	1.39E+09	4.42E+09	2.62E+04	6.21E+08	3.24E+08	7.88E+06	1.19E+05
	avg	2.79E+04	2.24E+04	1.45E+08	1.26E+09	5.86E+09	3.19E+04	1.21E+09	1.14E+08	3.96E+06	7.72E+04
F14	min	1.46E+03	1.48E+03	2.61E+05	1.32E+05	1.22E+06	4.46E+03	2.45E+05	6.53E+04	4.47E+04	7.05E+03
	std	3.25E+01	3.02E+01	9.54E+05	3.36E+06	6.58E+06	1.98E+05	5.48E+05	9.98E+05	3.30E+05	7.92E+04
	avg	1.54E+03	1.52E+03	1.59E+06	3.68E+06	6.56E+06	1.41E+05	1.11E+06	9.21E+05	4.67E+05	5.81E+04
F15	min	2.09E+03	1.90E+03	2.89E+04	8.49E+06	1.12E+07	2.62E+03	4.54E+06	3.21E+03	3.15E+03	2.30E+03
	std	1.18E+04	1.11E+04	1.03E+07	3.19E+08	7.30E+08	1.02E+04	6.34E+07	3.56E+04	1.67E+04	2.24E+04
	avg	7.22E+03	9.05E+03	5.61E+06	2.78E+08	5.64E+08	1.16E+04	9.18E+07	5.35E+04	2.40E+04	1.95E+04
F16	min	2.24E+03	1.99E+03	2.92E+03	3.85E+03	4.02E+03	2.34E+03	3.58E+03	2.25E+03	2.54E+03	1.98E+03
	std	2.76E+02	2.09E+02	2.86E+02	8.91E+02	9.32E+02	3.14E+02	2.72E+02	4.09E+02	4.09E+02	4.59E+02
	avg	2.64E+03	2.46E+03	3.34E+03	5.11E+03	5.68E+03	2.85E+03	4.24E+03	3.10E+03	3.35E+03	2.76E+03
F17	min	1.85E+03	1.75E+03	2.00E+03	2.40E+03	2.79E+03	2.31E+03	2.43E+03	1.88E+03	2.00E+03	1.82E+03
	std	1.49E+02	7.76E+01	3.28E+02	1.11E+03	1.62E+03	1.80E+02	2.21E+02	3.05E+02	2.12E+02	2.57E+02
	avg	2.07E+03	1.87E+03	2.46E+03	3.46E+03	3.82E+03	2.60E+03	2.92E+03	2.46E+03	2.52E+03	2.32E+03
F18	min	2.38E+03	2.21E+03	4.73E+04	9.55E+05	6.44E+06	1.23E+05	9.80E+05	8.89E+04	1.70E+05	8.75E+04
	std	1.47E+03	1.13E+04	1.23E+07	2.89E+07	6.30E+07	1.79E+06	1.24E+07	5.62E+06	5.16E+06	5.97E+05
	avg	3.70E+03	1.55E+04	7.48E+06	3.27E+07	7.37E+07	1.65E+06	1.78E+07	5.36E+06	3.45E+06	6.30E+05
F19	min	1.99E+03	1.98E+03	6.66E+05	3.26E+07	5.60E+07	2.15E+03	6.16E+07	2.64E+03	3.54E+03	2.27E+03
	std	4.18E+03	9.79E+03	8.05E+07	3.74E+08	6.11E+08	1.42E+04	6.60E+07	4.00E+07	7.07E+06	1.43E+04
	avg	3.54E+03	6.35E+03	3.13E+07	4.62E+08	7.42E+08	1.40E+04	1.25E+08	9.25E+06	2.22E+06	1.32E+04
F20	min	2.22E+03	2.06E+03	2.38E+03	2.71E+03	2.62E+03	2.43E+03	2.76E+03	2.34E+03	2.32E+03	2.30E+03
	std	1.22E+02	1.41E+02	2.22E+02	2.57E+02	2.23E+02	1.74E+02	1.51E+02	2.37E+02	2.22E+02	2.90E+02
	avg	2.43E+03	2.27E+03	2.76E+03	3.08E+03	3.02E+03	2.72E+03	3.01E+03	2.73E+03	2.68E+03	2.66E+03
F21	min	2.34E+03	2.35E+03	2.40E+03	2.64E+03	2.63E+03	2.46E+03	2.57E+03	2.41E+03	2.42E+03	2.37E+03
	std	2.52E+01	2.01E+01	3.28E+01	6.01E+01	6.06E+01	3.42E+01	2.84E+01	5.74E+01	5.07E+01	3.23E+01
	avg	2.38E+03	2.39E+03	2.45E+03	2.73E+03	2.74E+03	2.52E+03	2.62E+03	2.50E+03	2.51E+03	2.41E+03
F22	min	2.32E+03	2.30E+03	2.61E+03	8.27E+03	8.59E+03	2.30E+03	6.02E+03	2.74E+03	2.38E+03	2.33E+03
	std	2.21E+03	2.23E+03	2.16E+03	7.08E+02	5.43E+02	2.40E+03	1.14E+03	1.39E+03	2.33E+03	2.33E+03
	avg	5.60E+03	3.54E+03	4.54E+03	9.90E+03	9.99E+03	5.02E+03	1.01E+04	6.86E+03	4.19E+03	4.38E+03
F23	min	2.72E+03	2.69E+03	2.75E+03	3.07E+03	3.25E+03	2.82E+03	3.01E+03	2.76E+03	2.89E+03	2.75E+03
	std	5.30E+01	2.52E+01	2.97E+01	1.83E+02	1.72E+02	5.20E+01	5.95E+01	3.79E+01	9.63E+01	4.89E+01
	avg	2.78E+03	2.72E+03	2.80E+03	3.45E+03	3.51E+03	2.90E+03	3.10E+03	2.83E+03	3.03E+03	2.82E+03
F24	min	2.89E+03	2.86E+03	2.90E+03	3.23E+03	3.39E+03	2.93E+03	3.16E+03	2.94E+03	3.06E+03	2.90E+03
	std	4.48E+01	1.50E+01	3.22E+01	1.98E+02	1.59E+02	6.56E+01	4.37E+01	3.30E+01	8.44E+01	1.99E+02
	avg	2.94E+03	2.89E+03	2.97E+03	3.65E+03	3.69E+03	3.07E+03	3.26E+03	2.99E+03	3.20E+03	3.09E+03
F25	min	2.89E+03	2.89E+03	2.96E+03	3.64E+03	3.47E+03	2.88E+03	3.55E+03	2.95E+03	2.92E+03	2.90E+03
	std	2.60E+01	1.45E+01	3.91E+01	3.47E+02	5.62E+02	1.22E+01	2.15E+02	3.99E+02	3.13E+01	2.34E+01
	avg	2.92E+03	2.90E+03	3.04E+03	4.48E+03	4.79E+03	2.90E+03	3.87E+03	3.38E+03	2.97E+03	2.95E+03
F26	min	2.83E+03	2.80E+03	4.59E+03	8.56E+03	7.95E+03	2.91E+03	7.50E+03	5.01E+03	3.74E+03	4.60E+03
	std	6.22E+02	4.31E+02	3.35E+02	9.89E+02	1.30E+03	1.46E+03	5.31E+02	6.42E+02	1.49E+03	6.14E+02
	avg	4.89E+03	4.42E+03	5.08E+03	1.04E+04	1.09E+04	6.05E+03	8.38E+03	5.86E+03	6.41E+03	5.54E+03
F27	min	3.21E+03	3.20E+03	3.23E+03	3.38E+03	3.58E+03	3.23E+03	3.44E+03	3.22E+03	3.24E+03	3.20E+03
	std	2.61E+01	1.42E+01	3.44E+01	3.04E+02	4.27E+02	3.98E+01	7.25E+01	2.12E+01	5.93E+01	1.95E+02
	avg	3.24E+03	3.22E+03	3.28E+03	3.88E+03	4.36E+03	3.29E+03	3.58E+03	3.25E+03	3.33E+03	3.42E+03
F28	min	3.23E+03	3.22E+03	3.40E+03	4.79E+03	5.72E+03	3.21E+03	4.07E+03	3.28E+03	3.29E+03	3.29E+03
	std	4.24E+01	2.81E+01	8.05E+01	5.42E+02	6.12E+02	3.74E+01	3.73E+02	1.12E+03	1.27E+02	4.05E+01
	avg	3.30E+03	3.26E+03	3.54E+03	6.12E+03	6.74E+03	3.26E+03	4.76E+03	4.45E+03	3.44E+03	3.35E+03
F29	min	3.48E+03	3.47E+03	3.78E+03	4.73E+03	5.13E+03	3.68E+03	4.66E+03	3.76E+03	3.85E+03	3.72E+03
	std	2.27E+02	1.40E+02	1.97E+02	1.77E+03	1.51E+03	3.70E+02	4.29E+02	1.87E+02	2.49E+02	7.09E+02
	avg	3.85E+03	3.68E+03	4.18E+03	6.72E+03	7.36E+03	4.14E+03	5.44E+03	4.16E+03	4.32E+03	4.40E+03
F30	min	8.84E+03	7.43E+03	5.86E+06	6.25E+07	9.40E+07	6.81E+03	1.57E+08	2.49E+04	3.36E+04	9.55E+03
	std	3.80E+04	6.91E+03	4.25E+07	3.69E+08	5.35E+08	1.51E+04	8.25E+07	7.15E+05	9.29E+06	3.41E+05
	avg	3.91E+04	1.58E+04	3.66E+07	4.97E+08	7.06E+08	2.08E+04	2.53E+08	6.46E+05	4.49E+06	2.29E+05

**Table 5:** Ranking of benchmark test function results.

Function		RBMO	CLD-RBMO	GWO	WOA	HHO	SSA	SCA	MFO	DBO	HBA
F1	avg	1.109E+06	4.674E+03	4.603E+09	4.299E+10	5.507E+10	1.466E+05	2.346E+10	1.089E+10	5.898E+08	9.051E+07
	std	6.924E+05	3.247E+03	2.191E+09	7.941E+09	8.152E+09	1.167E+05	3.904E+09	9.124E+09	4.805E+08	1.667E+08
	rank	3	1	6	9	10	2	8	7	5	4
F3	avg	8.914E+03	5.403E+03	9.666E+04	2.752E+05	9.197E+04	5.499E+04	1.014E+05	2.170E+05	8.960E+04	5.776E+04
	std	3.230E+03	2.646E+03	1.851E+04	6.283E+04	3.795E+03	9.065E+03	2.010E+04	7.244E+04	2.192E+04	9.724E+03
	rank	2	1	7	10	6	3	8	9	5	4
F4	avg	5.290E+02	5.046E+02	7.264E+02	1.046E+04	1.427E+04	5.062E+02	4.220E+03	9.470E+02	6.184E+02	5.430E+02
	std	3.482E+01	3.201E+01	1.393E+02	3.064E+03	3.067E+03	2.320E+01	1.556E+03	6.390E+02	1.016E+02	3.612E+01
	rank	3	1	6	9	10	2	8	7	5	4
F5	avg	5.851E+02	5.896E+02	6.897E+02	9.309E+02	9.156E+02	7.495E+02	8.428E+02	7.069E+02	7.057E+02	6.296E+02
	std	2.095E+01	2.130E+01	2.519E+01	3.124E+01	4.705E+01	6.977E+01	2.486E+01	4.346E+01	4.818E+01	2.811E+01
	rank	1	2	4	10	9	7	8	6	5	3
F6	avg	6.048E+02	6.007E+02	6.162E+02	6.866E+02	6.900E+02	6.517E+02	6.692E+02	6.378E+02	6.424E+02	6.248E+02
	std	2.313E+00	4.600E-01	5.066E+00	1.036E+01	8.126E+00	1.310E+01	5.958E+00	1.031E+01	1.166E+01	7.142E+00
	rank	2	1	3	9	10	7	8	5	6	4
F7	avg	8.330E+02	8.184E+02	9.239E+02	1.449E+03	1.463E+03	1.241E+03	1.243E+03	1.127E+03	1.054E+03	9.715E+02
	std	2.927E+01	2.432E+01	4.292E+01	6.947E+01	4.775E+01	9.583E+01	4.910E+01	1.397E+02	8.564E+01	6.446E+01
	rank	2	1	3	9	10	7	8	6	5	4
F8	avg	8.854E+02	8.943E+02	9.523E+02	1.135E+03	1.148E+03	9.842E+02	1.107E+03	1.019E+03	9.572E+02	9.189E+02
	std	2.412E+01	2.378E+01	2.442E+01	2.969E+01	2.950E+01	2.139E+01	2.408E+01	4.683E+01	3.360E+01	3.098E+01
	rank	1	2	4	9	10	6	8	7	5	3
F9	avg	1.146E+03	9.377E+02	2.323E+03	1.146E+04	1.157E+04	5.268E+03	9.559E+03	7.273E+03	8.020E+03	4.016E+03
	std	1.970E+02	6.311E+01	8.254E+02	1.838E+03	1.970E+03	4.067E+02	2.337E+03	1.906E+03	1.539E+03	1.425E+03
	rank	2	1	3	9	10	5	8	6	7	4
F10	avg	5.431E+03	5.778E+03	5.770E+03	8.763E+03	8.778E+03	5.567E+03	8.779E+03	5.661E+03	7.207E+03	6.354E+03
	std	7.203E+02	5.963E+02	5.979E+02	4.302E+02	5.196E+02	7.831E+02	4.191E+02	7.013E+02	1.256E+03	1.247E+03
	rank	1	5	4	8	9	2	10	3	7	6
F11	avg	1.247E+03	1.175E+03	6.125E+03	1.489E+04	1.224E+04	1.299E+03	4.351E+03	6.811E+03	2.196E+03	1.369E+03
	std	3.646E+01	3.059E+01	1.760E+03	4.810E+03	2.836E+03	6.021E+01	1.019E+03	5.448E+03	7.758E+02	8.059E+01
	rank	2	1	7	10	9	3	6	8	5	4
F12	avg	6.694E+05	6.585E+05	2.989E+08	7.796E+09	1.091E+10	2.387E+06	2.991E+09	1.687E+08	8.546E+07	4.258E+06
	std	8.058E+05	6.828E+05	1.908E+08	3.805E+09	3.822E+09	1.753E+06	8.734E+08	2.209E+08	8.230E+07	3.207E+06
	rank	2	1	7	9	10	3	8	6	5	4
F13	avg	2.787E+04	2.238E+04	1.451E+08	1.261E+09	5.858E+09	3.193E+04	1.211E+09	1.140E+08	3.956E+06	7.721E+04
	std	2.021E+04	2.150E+04	2.353E+08	1.394E+09	4.423E+09	2.619E+04	6.206E+08	3.240E+08	7.882E+06	1.188E+05
	rank	2	1	7	9	10	3	8	6	5	4
F14	avg	1.545E+03	1.519E+03	1.585E+06	3.676E+06	6.556E+06	1.406E+05	1.108E+06	9.213E+05	4.674E+05	5.809E+04
	std	3.254E+01	3.022E+01	9.539E+05	3.361E+06	6.578E+06	1.979E+05	5.483E+05	9.980E+05	3.300E+05	7.919E+04
	rank	2	1	8	9	10	4	7	6	5	3
F15	avg	7.219E+03	9.050E+03	5.608E+06	2.783E+08	5.638E+08	1.155E+04	9.180E+07	5.348E+04	2.403E+04	1.946E+04
	std	1.176E+04	1.109E+04	1.035E+07	3.186E+08	7.305E+08	1.021E+04	6.335E+07	3.564E+04	1.672E+04	2.238E+04
	rank	1	2	7	9	10	3	8	6	5	4
F16	avg	2.637E+03	2.457E+03	3.338E+03	5.113E+03	5.676E+03	2.850E+03	4.237E+03	3.103E+03	3.348E+03	2.756E+03
	std	2.757E+02	2.092E+02	2.856E+02	8.906E+02	9.324E+02	3.137E+02	2.718E+02	4.086E+02	4.090E+02	4.586E+02
	rank	2	1	6	9	10	4	8	5	7	3
F17	avg	2.069E+03	1.874E+03	2.462E+03	3.460E+03	3.817E+03	2.597E+03	2.917E+03	2.465E+03	2.518E+03	2.323E+03
	std	1.493E+02	7.758E+01	3.279E+02	1.114E+03	1.617E+03	1.803E+02	2.206E+02	3.045E+02	2.119E+02	2.569E+02
	rank	2	1	4	9	10	7	8	5	6	3
F18	avg	3.704E+03	1.548E+04	7.476E+06	3.267E+07	7.374E+07	1.653E+06	1.775E+07	5.359E+06	3.448E+06	6.301E+05
	std	1.471E+03	1.126E+04	1.225E+07	2.890E+07	6.304E+07	1.795E+06	1.238E+07	5.623E+06	5.158E+06	5.973E+05
	rank	1	2	7	9	10	4	8	6	5	3
F19	avg	3.536E+03	6.355E+03	3.130E+07	4.619E+08	7.421E+08	1.395E+04	1.251E+08	9.254E+06	2.216E+06	1.324E+04
	std	4.178E+03	9.788E+03	8.050E+07	3.743E+08	6.110E+08	1.420E+04	6.599E+07	3.996E+07	7.071E+06	1.431E+04
	rank	1	2	7	9	10	4	8	6	5	3
F20	avg	2.434E+03	2.266E+03	2.758E+03	3.084E+03	3.020E+03	2.720E+03	3.014E+03	2.727E+03	2.679E+03	2.658E+03
	std	1.224E+02	1.407E+02	2.219E+02	2.569E+02	2.229E+02	1.739E+02	1.512E+02	2.365E+02	2.223E+02	2.901E+02
	rank	2	1	7	10	9	5	8	6	4	3
F21	avg	2.377E+03	2.386E+03	2.447E+03	2.735E+03	2.737E+03	2.519E+03	2.621E+03	2.501E+03	2.511E+03	2.415E+03
	std	2.525E+01	2.014E+01	3.276E+01	6.006E+01	6.060E+01	3.416E+01	2.841E+01	5.744E+01	5.071E+01	3.232E+01
	rank	1	2	4	9	10	7	8	5	6	3
F22	avg	5.595E+03	3.535E+03	4.543E+03	9.899E+03	9.990E+03	5.022E+03	1.012E+04	6.857E+03	4.191E+03	4.384E+03
	std	2.211E+03	2.230E+03	2.161E+03	7.078E+02	5.435E+02	2.398E+03	1.140E+03	1.390E+03	2.330E+03	2.326E+03
	rank	6	1	4	8	9	5	10	7	2	3
F23	avg	2.776E+03	2.724E+03	2.802E+03	3.451E+03	3.512E+03	2.898E+03	3.097E+03	2.827E+03	3.031E+03	2.819E+03
	std	5.297E+01	2.522E+01	2.970E+01	1.828E+02	1.722E+02	5.203E+01	5.953E+01	3.791E+01	9.626E+01	4.893E+01
	rank	2	1	3	9	10	6	8	5	7	4
F24	avg	2.940E+03	2.890E+03	2.972E+03	3.647E+03	3.688E+03	3.068E+03	3.264E+03	2.988E+03	3.204E+03	3.090E+03
	std	4.483E+01	1.498E+01	3.224E+01	1.985E+02	1.588E+02	6.559E+01	4.371E+01	3.305E+01	8.436E+01	1.989E+02
	rank	2	1	3	9	10	5	8	4	7	6
F25	avg	2.915E+03	2.898E+03	3.039E+03	4.477E+03	4.790E+03	2.897E+03	3.872E+03	3.377E+03	2.966E+03	2.946E+03
	std	2.597E+01	1.452E+01	3.914E+01	3.473E+02	5.617E+02	1.224E+01	2.152E+02	3.988E+02	3.134E+01	2.341E+01
	rank	3	2	6	9	10	1	8	7	5	4
F26	avg	4.888E+03	4.418E+03	5.085E+03	1.041E+04	1.094E+04	6.054E+03	8.383E+03	5.861E+03	6.407E+03	5.538E+03
	std	6.217E+02	4.313E+02	3.354E+02	9.887E+02	1.304E+03	1.462E+03	5.311E+02	6.423E+02	1.487E+03	6.140E+02
	rank	2	1	3	9	10	6	8	5	7	4
F27	avg	3.240E+03	3.220E+03	3.275E+03	3.876E+03	4.362E+03	3.288E+03	3.584E+03	3.252E+03	3.333E+03	3.424E+03
	std	2.606E+01	1.415E+01	3.443E+01	3.041E+02	4.267E+02	3.981E+01	7.252E+01	2.120E+01	5.934E+01	1.954E+02
	rank	2	1	4	9	10	5	8	3	6	7
F28	avg	3.297E+03	3.261E+03	3.544E+03	6.124E+03	6.741E+03	3.262E+03	4.758E+03	4.445E+03	3.438E+03	3.348E+03
	std	4.239E+01	2.808E+01	8.054E+01	5.425E+02	6.119E+02	3.743E+01	3.734E+02	1.123E+03	1.267E+02	4.049E+01
	rank	3	1	6	9	10	2	8	7	5	4
F29	avg	3.851E+03	3.678E+03	4.176E+03	6.723E+03	7.355E+03	4.142E+03	5.438E+03	4.163E+03	4.321E+03	4.398E+03
	std	2.275E+02	1.397E+02	1.969E+02	1.773E+03	1.512E+03	3.697E+02	4.286E+02	1.866E+02	2.493E+02	7.093E+02
	rank	2	1	5	9	10	3	8	4	6	7
F30	avg	3.906E+04	1.580E+04	3.661E+07	4.968E+08	7.056E+08	2.083E+04	2.535E+08	6.465E+05	4.486E+06	2.290E+05
	std	3.796E+04	6.915E+03	4.252E+07	3.690E+08	5.352E+08	1.509E+04	8.252E+07	7.150E+05	9.289E+06	3.411E+05
	rank	3	1	7	9	10	2	8	5	6	4
Mean Rank		2.07	1.38	5.24	9.07	9.69	4.24	8.03	5.79	5.48	4
Total Rank		2	1	5	9	10	4	8	7	6	3

**Table 6:** Results of the Wilcoxon rank-sum test.

CLD-RBMO		GWO	WOA	HHO	SSA	SCA	MFO	DBO	HBA
F1	6.80E-08	6.80E-08	6.80E-08	6.80E-08	2.96E-07	6.80E-08	6.80E-08	6.80E-08	6.80E-08
F3	1.48E-03	6.80E-08	6.80E-08	6.80E-08	6.80E-08	6.80E-08	6.80E-08	6.80E-08	6.80E-08
F4	3.06E-03	2.22E-07	6.80E-08	6.80E-08	2.23E-02	6.80E-08	1.05E-06	2.00E-04	1.26E-01
F5	2.62E-01	7.90E-08	6.80E-08	6.80E-08	9.17E-08	6.80E-08	9.17E-08	9.17E-08	1.81E-05
F6	6.80E-08	2.96E-07	6.80E-08	6.80E-08	6.80E-08	6.80E-08	6.80E-08	6.80E-08	6.80E-08
F7	1.14E-01	1.38E-06	6.80E-08	6.80E-08	6.80E-08	6.80E-08	6.80E-08	6.80E-08	9.17E-08
F8	2.73E-01	2.56E-07	6.80E-08	6.80E-08	6.80E-08	6.80E-08	6.80E-08	2.96E-07	1.63E-03
F9	3.94E-07	1.43E-07	6.80E-08	6.80E-08	6.80E-08	6.80E-08	6.80E-08	6.80E-08	7.90E-08
F10	1.56E-01	1.81E-01	6.80E-08	6.80E-08	7.76E-01	6.80E-08	3.65E-01	2.92E-05	1.06E-02
F11	3.99E-06	6.80E-08	6.80E-08	6.80E-08	1.63E-03	6.80E-08	6.80E-08	6.80E-08	4.54E-06
F12	7.76E-01	6.80E-08	6.80E-08	6.80E-08	1.44E-04	6.80E-08	6.80E-08	7.90E-08	5.17E-06
F13	3.23E-01	6.80E-08	6.80E-08	6.80E-08	7.76E-01	6.80E-08	1.05E-06	3.94E-07	3.37E-02
F14	3.34E-03	6.80E-08	6.80E-08	6.80E-08	6.80E-08	6.80E-08	6.80E-08	6.80E-08	6.80E-08
F15	1.99E-01	9.17E-08	6.80E-08	6.80E-08	5.09E-04	6.80E-08	1.80E-06	2.60E-05	4.16E-04
F16	4.68E-02	1.20E-06	6.80E-08	6.80E-08	4.11E-02	6.80E-08	4.60E-04	6.67E-06	4.90E-01
F17	2.30E-05	6.61E-05	6.80E-08	6.80E-08	1.06E-07	6.80E-08	1.04E-04	1.38E-06	1.01E-03
F18	1.41E-05	6.80E-08	6.80E-08	6.80E-08	6.80E-08	6.80E-08	6.80E-08	6.80E-08	6.80E-08
F19	2.50E-01	6.80E-08	6.80E-08	6.80E-08	2.92E-05	6.80E-08	3.42E-07	1.05E-06	1.81E-05
F20	5.63E-04	4.17E-05	6.80E-08	9.17E-08	7.58E-06	6.80E-08	1.04E-04	1.44E-04	1.79E-02
F21	1.02E-01	6.01E-07	6.80E-08	6.80E-08	6.80E-08	6.80E-08	1.43E-07	1.23E-07	3.75E-04
F22	3.38E-04	4.25E-01	7.90E-08	6.80E-08	2.62E-01	4.54E-07	6.01E-02	3.65E-01	1.08E-01
F23	5.25E-05	6.56E-03	6.80E-08	6.80E-08	3.07E-06	6.80E-08	3.05E-04	1.66E-07	3.64E-03
F24	1.60E-05	2.56E-03	6.80E-08	6.80E-08	1.58E-06	6.80E-08	8.29E-05	7.90E-08	1.95E-03
F25	3.06E-03	9.17E-08	6.80E-08	6.80E-08	3.97E-03	6.80E-08	1.23E-07	1.41E-05	3.75E-04
F26	4.60E-04	2.73E-01	6.80E-08	6.80E-08	2.75E-04	6.80E-08	1.60E-05	6.22E-04	2.14E-03
F27	9.05E-03	4.16E-04	6.80E-08	6.80E-08	7.41E-05	6.80E-08	4.99E-02	1.38E-06	1.16E-04
F28	8.36E-04	7.90E-08	6.80E-08	6.80E-08	4.16E-04	6.80E-08	6.92E-07	1.25E-05	2.30E-05
F29	1.23E-02	1.16E-04	6.80E-08	6.80E-08	1.14E-02	6.80E-08	1.79E-04	4.54E-06	5.09E-04
F30	1.23E-02	6.80E-08	6.80E-08	6.80E-08	8.59E-02	6.80E-08	8.60E-06	9.13E-07	3.60E-02
+/−	20/9	26/3	29/0	29/0	25/4	29/0	27/2	28/1	26/3

**Table 7:** Results of Ablation Study on Benchmark Functions.

Function		CLD_RBMO	LD_RBMO	CD_RBMO	CL_RBMO
F1	min	3.604E+02	2.915E+02	2.031E+02	3.450E+02
	std	3.186E+03	5.751E+03	5.439E+03	4.667E+03
	avg	4.238E+03	5.736E+03	4.905E+03	4.935E+03
F3	min	4.968E+02	6.472E+02	7.667E+02	1.013E+04
	std	7.430E+02	1.302E+03	7.637E+02	7.876E+03
	avg	1.172E+03	2.359E+03	1.627E+03	1.795E+04
F4	min	4.718E+02	4.003E+02	4.194E+02	4.687E+02
	std	2.357E+01	3.508E+01	2.361E+01	1.871E+01
	avg	5.059E+02	4.976E+02	4.961E+02	5.086E+02
F5	min	5.308E+02	5.332E+02	5.608E+02	5.311E+02
	std	1.397E+01	1.785E+01	1.981E+01	1.590E+01
	avg	5.592E+02	5.580E+02	5.973E+02	5.648E+02
F6	min	6.000E+02	6.000E+02	6.000E+02	6.001E+02
	std	7.343E-01	1.034E+00	1.175E-01	3.770E-01
	avg	6.004E+02	6.007E+02	6.002E+02	6.005E+02
F7	min	7.574E+02	7.702E+02	7.669E+02	7.631E+02
	std	2.079E+01	2.676E+01	2.706E+01	1.769E+01
	avg	7.907E+02	8.126E+02	8.269E+02	7.908E+02
F8	min	8.358E+02	8.249E+02	8.627E+02	8.309E+02
	std	1.987E+01	2.237E+01	2.563E+01	1.695E+01
	avg	8.718E+02	8.586E+02	8.921E+02	8.573E+02
F9	min	9.040E+02	9.025E+02	9.036E+02	9.044E+02
	std	1.721E+01	8.701E+01	7.524E+00	1.500E+02
	avg	9.163E+02	9.539E+02	9.121E+02	9.790E+02
F10	min	3.791E+03	4.057E+03	4.359E+03	4.041E+03
	std	5.962E+02	7.439E+02	5.501E+02	4.143E+02
	avg	5.050E+03	5.534E+03	5.625E+03	4.872E+03
F11	min	1.119E+03	1.122E+03	1.120E+03	1.132E+03
	std	3.582E+01	3.579E+01	3.279E+01	3.863E+01
	avg	1.178E+03	1.190E+03	1.184E+03	1.194E+03
F12	min	1.845E+04	1.019E+04	1.996E+04	1.716E+04
	std	2.183E+05	1.389E+05	3.053E+05	7.644E+04
	avg	1.426E+05	1.384E+05	2.505E+05	1.148E+05
F13	min	1.466E+03	1.351E+03	1.515E+03	1.484E+03
	std	1.607E+04	2.191E+04	2.366E+04	2.549E+04
	avg	1.602E+04	2.117E+04	2.626E+04	2.558E+04
F14	min	1.462E+03	1.464E+03	1.463E+03	1.608E+03
	std	3.650E+01	3.096E+01	3.486E+01	1.894E+04
	avg	1.503E+03	1.503E+03	1.518E+03	1.546E+04
F15	min	1.777E+03	1.732E+03	1.907E+03	1.784E+03
	std	9.520E+03	3.082E+03	1.151E+04	8.369E+03
	avg	5.556E+03	3.435E+03	6.136E+03	9.476E+03
F16	min	1.708E+03	1.897E+03	1.845E+03	1.841E+03
	std	2.980E+02	2.406E+02	2.928E+02	2.820E+02
	avg	2.329E+03	2.338E+03	2.379E+03	2.411E+03
F17	min	1.751E+03	1.744E+03	1.750E+03	1.813E+03
	std	1.174E+02	1.200E+02	9.930E+01	1.127E+02
	avg	1.900E+03	1.907E+03	1.871E+03	1.915E+03
F18	min	2.504E+03	2.221E+03	4.338E+03	5.149E+04
	std	1.575E+04	2.200E+04	1.320E+04	2.718E+05
	avg	1.757E+04	2.346E+04	1.740E+04	2.403E+05
F19	min	1.989E+03	2.015E+03	1.990E+03	2.007E+03
	std	1.280E+04	1.218E+04	1.607E+04	1.249E+04
	avg	7.279E+03	7.816E+03	7.531E+03	1.644E+04
F20	min	2.032E+03	2.025E+03	2.039E+03	2.090E+03
	std	1.116E+02	1.526E+02	9.206E+01	9.715E+01
	avg	2.224E+03	2.215E+03	2.164E+03	2.284E+03
F21	min	2.341E+03	2.342E+03	2.350E+03	2.334E+03
	std	1.998E+01	1.871E+01	1.845E+01	1.669E+01
	avg	2.371E+03	2.365E+03	2.385E+03	2.362E+03
F22	min	2.300E+03	2.300E+03	2.300E+03	2.300E+03
	std	1.941E+03	2.249E+03	2.542E+03	1.491E+03
	avg	3.526E+03	4.259E+03	4.745E+03	3.139E+03
F23	min	2.686E+03	2.690E+03	2.698E+03	2.694E+03
	std	1.889E+01	1.816E+01	2.702E+01	1.972E+01
	avg	2.720E+03	2.725E+03	2.742E+03	2.719E+03
F24	min	2.836E+03	2.857E+03	2.837E+03	2.849E+03
	std	1.744E+01	2.762E+01	2.322E+01	1.233E+01
	avg	2.872E+03	2.892E+03	2.898E+03	2.879E+03
F25	min	2.884E+03	2.884E+03	2.887E+03	2.884E+03
	std	1.757E+01	3.903E+00	5.002E+00	1.468E+01
	avg	2.897E+03	2.889E+03	2.890E+03	2.896E+03
F26	min	3.911E+03	2.900E+03	4.101E+03	4.033E+03
	std	1.788E+02	5.395E+02	2.340E+02	1.852E+02
	avg	4.191E+03	4.350E+03	4.493E+03	4.297E+03
F27	min	3.194E+03	3.210E+03	3.191E+03	3.203E+03
	std	1.031E+01	2.207E+01	1.292E+01	1.618E+01
	avg	3.216E+03	3.233E+03	3.213E+03	3.229E+03
F28	min	3.203E+03	3.210E+03	3.209E+03	3.198E+03
	std	1.948E+01	4.068E+01	1.873E+01	2.187E+01
	avg	3.236E+03	3.253E+03	3.233E+03	3.242E+03
F29	min	3.363E+03	3.361E+03	3.413E+03	3.455E+03
	std	1.418E+02	1.413E+02	9.760E+01	1.806E+02
	avg	3.558E+03	3.592E+03	3.654E+03	3.687E+03
F30	min	5.561E+03	6.737E+03	6.827E+03	7.866E+03
	std	3.930E+03	5.720E+03	9.882E+03	4.889E+03
	avg	1.122E+04	1.283E+04	1.778E+04	1.303E+04

**Table 8:** Ranking of Ablation Variants on Benchmark Functions.

Function		CLD_RBMO	LD_RBMO	CD_RBMO	CL_RBMO
F1	avg	4.238E+03	5.736E+03	4.905E+03	4.935E+03
	std	3.186E+03	5.751E+03	5.439E+03	4.667E+03
	rank	1	4	2	3
F3	avg	1.172E+03	2.359E+03	1.627E+03	1.795E+04
	std	7.430E+02	1.302E+03	7.637E+02	7.876E+03
	rank	1	3	2	4
F4	avg	5.059E+02	4.976E+02	4.961E+02	5.086E+02
	std	2.357E+01	3.508E+01	2.361E+01	1.871E+01
	rank	3	2	1	4
F5	avg	5.592E+02	5.580E+02	5.973E+02	5.648E+02
	std	1.397E+01	1.785E+01	1.981E+01	1.590E+01
	rank	2	1	4	3
F6	avg	6.004E+02	6.007E+02	6.002E+02	6.005E+02
	std	7.343E-01	1.034E+00	1.175E-01	3.770E-01
	rank	2	4	1	3
F7	avg	7.907E+02	8.126E+02	8.269E+02	7.908E+02
	std	2.079E+01	2.676E+01	2.706E+01	1.769E+01
	rank	1	3	4	2
F8	avg	8.718E+02	8.586E+02	8.921E+02	8.573E+02
	std	1.987E+01	2.237E+01	2.563E+01	1.695E+01
	rank	3	2	4	1
F9	avg	9.163E+02	9.539E+02	9.121E+02	9.790E+02
	std	1.721E+01	8.701E+01	7.524E+00	1.500E+02
	rank	2	3	1	4
F10	avg	5.050E+03	5.534E+03	5.625E+03	4.872E+03
	std	5.962E+02	7.439E+02	5.501E+02	4.143E+02
	rank	2	3	4	1
F11	avg	1.178E+03	1.190E+03	1.184E+03	1.194E+03
	std	3.582E+01	3.579E+01	3.279E+01	3.863E+01
	rank	1	3	2	4
F12	avg	1.426E+05	1.384E+05	2.505E+05	1.148E+05
	std	2.183E+05	1.389E+05	3.053E+05	7.644E+04
	rank	3	2	4	1
F13	avg	1.602E+04	2.117E+04	2.626E+04	2.558E+04
	std	1.607E+04	2.191E+04	2.366E+04	2.549E+04
	rank	1	2	4	3
F14	avg	1.503E+03	1.503E+03	1.518E+03	1.546E+04
	std	3.650E+01	3.096E+01	3.486E+01	1.894E+04
	rank	1	2	3	4
F15	avg	5.556E+03	3.435E+03	6.136E+03	9.476E+03
	std	9.520E+03	3.082E+03	1.151E+04	8.369E+03
	rank	2	1	3	4
F16	avg	2.329E+03	2.338E+03	2.379E+03	2.411E+03
	std	2.980E+02	2.406E+02	2.928E+02	2.820E+02
	rank	2	1	3	4
F17	avg	1.900E+03	1.907E+03	1.871E+03	1.915E+03
	std	1.174E+02	1.200E+02	9.930E+01	1.127E+02
	rank	2	3	1	4
F18	avg	1.757E+04	2.346E+04	1.740E+04	2.403E+05
	std	1.575E+04	2.200E+04	1.320E+04	2.718E+05
	rank	2	3	1	4
F19	avg	7.279E+03	7.816E+03	7.531E+03	1.644E+04
	std	1.280E+04	1.218E+04	1.607E+04	1.249E+04
	rank	1	2	3	4
F20	avg	2.224E+03	2.215E+03	2.164E+03	2.284E+03
	std	1.116E+02	1.526E+02	9.206E+01	9.715E+01
	rank	3	2	1	4
F21	avg	2.371E+03	2.365E+03	2.385E+03	2.362E+03
	std	1.998E+01	1.871E+01	1.845E+01	1.669E+01
	rank	3	2	4	1
F22	avg	3.526E+03	4.259E+03	4.745E+03	3.139E+03
	std	1.941E+03	2.249E+03	2.542E+03	1.491E+03
	rank	2	3	4	1
F23	avg	2.720E+03	2.725E+03	2.742E+03	2.719E+03
	std	1.889E+01	1.816E+01	2.702E+01	1.972E+01
	rank	2	3	4	1
F24	avg	2.872E+03	2.892E+03	2.898E+03	2.879E+03
	std	1.744E+01	2.762E+01	2.322E+01	1.233E+01
	rank	1	3	4	2
F25	avg	2.897E+03	2.889E+03	2.890E+03	2.896E+03
	std	1.757E+01	3.903E+00	5.002E+00	1.468E+01
	rank	4	1	2	3
F26	avg	4.191E+03	4.350E+03	4.493E+03	4.297E+03
	std	1.788E+02	5.395E+02	2.340E+02	1.852E+02
	rank	1	3	4	2
F27	avg	3.216E+03	3.233E+03	3.213E+03	3.229E+03
	std	1.031E+01	2.207E+01	1.292E+01	1.618E+01
	rank	2	4	1	3
F28	avg	3.236E+03	3.253E+03	3.233E+03	3.242E+03
	std	1.948E+01	4.068E+01	1.873E+01	2.187E+01
	rank	2	4	1	3
F29	avg	3.558E+03	3.592E+03	3.654E+03	3.687E+03
	std	1.418E+02	1.413E+02	9.760E+01	1.806E+02
	rank	1	2	3	4
F30	avg	1.122E+04	1.283E+04	1.778E+04	1.303E+04
	std	3.930E+03	5.720E+03	9.882E+03	4.889E+03
	rank	1	2	4	3
Mean Rank		1.86	2.52	2.72	2.90
Total Rank		1	2	3	4

**Table 9:** Sensitivity analysis results of the Logistic parameter α on benchmark functions.

α	3.6	3.65	3.6884	3.75	3.8
F1	4.111E+03	4.982E+03	3.506E+03	4.549E+03	4.741E+03
F3	1.270E+03	1.342E+03	1.286E+03	1.178E+03	1.349E+03
F6	6.002E+02	6.002E+02	6.001E+02	6.002E+02	6.003E+02
F9	9.302E+02	9.350E+02	9.145E+02	9.227E+02	9.285E+02
F23	2.710E+03	2.705E+03	2.710E+03	2.709E+03	2.711E+03
F26	4.231E+03	4.188E+03	4.189E+03	4.098E+03	4.100E+03

**Table 10:** Sensitivity analysis results of the chaos frequency $k_{\mathrm{freq}}$ on benchmark functions.

$k_{\mathrm{freq}}$	1	2	3	4	5
F1	4.097E+03	6.220E+03	4.095E+03	4.518E+03	5.030E+03
F3	1.188E+03	1.362E+03	1.151E+03	1.370E+03	1.842E+03
F6	6.010E+02	6.002E+02	6.002E+02	6.001E+02	6.001E+02
F9	9.609E+02	9.371E+02	9.196E+02	9.104E+02	9.187E+02
F23	2.712E+03	2.706E+03	2.715E+03	2.711E+03	2.719E+03
F26	3.830E+03	4.240E+03	4.150E+03	4.163E+03	4.281E+03

**Table 11:** Sensitivity analysis results of the Lévy distribution parameter *β* on benchmark functions.

β	1.3	1.5	1.6973	1.9	2.1
F1	5.267E+03	4.878E+03	3.879E+03	2.577E+03	2.755E+03
F3	1.226E+03	9.843E+02	1.236E+03	1.198E+03	1.433E+03
F6	6.003E+02	6.002E+02	6.002E+02	6.003E+02	6.003E+02
F9	9.331E+02	9.287E+02	9.369E+02	9.193E+02	9.514E+02
F23	2.711E+03	2.709E+03	2.709E+03	2.715E+03	2.732E+03
F26	4.066E+03	4.182E+03	4.220E+03	4.047E+03	4.304E+03

**Table 12:** Sensitivity analysis results of the scaling coefficient c on benchmark functions.

c	0.01	0.03	0.0523	0.08	0.12
F1	7.423E+03	5.088E+03	4.138E+03	4.157E+03	2.310E+03
F3	9.197E+02	9.785E+02	1.029E+03	1.754E+03	2.265E+03
F6	6.002E+02	6.001E+02	6.003E+02	6.002E+02	6.004E+02
F9	9.399E+02	9.367E+02	9.328E+02	9.306E+02	9.296E+02
F23	2.727E+03	2.711E+03	2.712E+03	2.711E+03	2.705E+03
F26	4.130E+03	4.223E+03	3.993E+03	4.135E+03	4.223E+03

**Table 13:** Sensitivity analysis results of the upper bound of scaling factor $F_{\max}$ on benchmark functions.

$F_{\max}$	0.6	0.9	1.163	1.4	1.8
F1	4.705E+03	6.644E+03	3.717E+03	3.480E+03	5.497E+03
F3	3.075E+03	1.318E+03	1.477E+03	1.373E+03	1.944E+03
F6	6.003E+02	6.002E+02	6.003E+02	6.002E+02	6.003E+02
F9	1.014E+03	9.364E+02	9.253E+02	9.248E+02	9.301E+02
F23	2.709E+03	2.711E+03	2.710E+03	2.712E+03	2.715E+03
F26	4.141E+03	4.254E+03	3.984E+03	4.098E+03	4.306E+03

**Table 14:** Results of the Welded Beam Design problem.

Algorithm	*X*_1_	*X*_2_	*X*_3_	*X*_4_	Optimal Solution
CLD-RBMO	1.9883230722E-01	3.3373652986E+00	9.1920243225E+00	1.9883230722E-01	1.6702177263E+00
RBMO	1.9883230959E-01	3.3373652714E+00	9.1920242585E+00	1.9883231008E-01	1.6702177375E+00
PSO	1.9882177568E-01	3.3375870448E+00	9.1919704436E+00	1.9883492420E-01	1.6702425966E+00
WOA	1.8229722219E-01	5.9748230938E+00	9.2196398553E+00	1.9870336727E-01	1.9798537671E+00
HHO	1.7478344144E-01	4.0825170079E+00	8.6834921802E+00	2.2280269857E-01	1.8208738784E+00
SSA	1.9882186943E-01	3.3375686057E+00	9.1920046760E+00	1.9883319144E-01	1.6702326997E+00
SCA	1.7142121603E-01	4.0389364869E+00	9.1903927249E+00	2.0202903913E-01	1.7424784548E+00
MFO	1.9883223812E-01	3.3373666718E+00	9.1920242105E+00	1.9883231231E-01	1.6702178261E+00
DBO	1.9791790718E-01	3.3354065691E+00	9.2630198167E+00	1.9850231543E-01	1.6778468754E+00
HBA	1.9883183371E-01	3.3373555078E+00	9.1921101553E+00	1.9883190769E-01	1.6702269153E+00

**Table 15:** Results of the 10-bar truss design problem.

	CLD-RBMO	RBMO	PSO	WOA	HHO	SSA	SCA	MFO	DBO	HBA
X1	3.512371E-03	3.490119E-03	3.354243E-03	3.333293E-03	3.440554E-03	3.457293E-03	4.292157E-03	3.683466E-03	3.649279E-03	3.526218E-03
X2	1.475168E-03	1.459280E-03	1.471736E-03	2.306764E-03	1.197302E-03	1.468748E-03	1.168516E-03	1.428645E-03	1.106693E-03	1.464477E-03
X3	3.514823E-03	3.535212E-03	3.559951E-03	3.923650E-03	3.541987E-03	3.602014E-03	4.125999E-03	3.374850E-03	3.539025E-03	3.542285E-03
X4	1.469742E-03	1.469361E-03	1.477460E-03	1.802371E-03	2.627480E-03	1.482604E-03	2.454733E-03	1.631465E-03	1.712050E-03	1.496323E-03
X5	6.454388E-05	6.450002E-05	6.450000E-05	2.555834E-03	1.390559E-03	6.450000E-05	1.171470E-04	6.450000E-05	6.450000E-05	6.476892E-05
X6	4.558150E-04	4.567550E-04	4.614952E-04	3.974758E-04	3.159578E-04	4.550059E-04	7.844540E-04	4.509194E-04	5.676800E-04	4.545075E-04
X7	2.373981E-03	2.362719E-03	2.359793E-03	2.840355E-03	3.255882E-03	2.361182E-03	1.510959E-03	2.121104E-03	2.017686E-03	2.335145E-03
X8	2.364424E-03	2.390528E-03	2.361610E-03	1.398474E-03	9.824249E-04	2.386027E-03	2.675444E-03	2.536795E-03	2.833177E-03	2.407765E-03
X9	1.245065E-03	1.254243E-03	1.270244E-03	6.851101E-04	2.254968E-03	1.187245E-03	1.380469E-03	1.305584E-03	1.455672E-03	1.199898E-03
X10	1.238450E-03	1.227262E-03	1.319882E-03	2.386150E-03	1.639997E-03	1.269376E-03	9.797810E-04	1.187768E-03	9.748057E-04	1.247737E-03
Best	5.244547E+02	5.244778E+02	5.250537E+02	6.245447E+02	6.082990E+02	5.247617E+02	5.623359E+02	5.255044E+02	5.303006E+02	5.247522E+02

## Data Availability

The data that support the findings of this study are available from the corresponding author upon request. There are no restrictions on data availability.

## References

[1] Wang X., Han Y., Chu C., Geng Z. (2026). Adaptive small-family population-guided swarm intelligence optimization algorithm. Sci. China Inf. Sci..

[2] Hu G., Huang F., Chen K., Wei G. (2024). MNEARO: A meta swarm intelligence optimization algorithm for engineering applications. Comput. Methods Appl. Mech. Eng..

[3] Yue Y., Cao L., Lu D., Hu Z., Xu M., Wang S., Li B., Ding H. (2023). Review and empirical analysis of sparrow search algorithm. Artif. Intell. Rev..

[4] Wang X., Cao L., Yue Y. (2025). Coverage optimization strategy for 3D wireless sensor network based on adaptive inertia weight Arctic Puffin Optimization Algorithm. J. Comput. Des. Eng..

[5] Wang Z., Yao L., Li M., Chen M., Zhao J., Chu F., Li W.J. (2024). A high-accuracy fault detection method using swarm intelligence optimization entropy. IEEE Trans. Instrum. Meas..

[6] Cao L., Yue Y., Chen Y., Chen C., Chen B. (2025). Sailfish Optimization Algorithm Integrated with the Osprey Optimization Algorithm and Cauchy Mutation and Its Engineering Applications. Symmetry.

[7] Yue Y., Cao L., Chen C., Chen Y., Chen B. (2025). Snake Optimization Algorithm Augmented by Adaptive t-Distribution Mixed Mutation and Its Application in Energy Storage System Capacity Optimization. Biomimetics.

[8] Liu B., Xu M., Gao L. (2024). Enhanced swarm intelligence optimization: Inspired by cellular coordination in immune systems. Knowl.-Based Syst..

[9] Liu Y., Huang H., Zhou J. (2024). A dual cluster head hierarchical routing protocol for wireless sensor networks based on hybrid swarm intelligence optimization. IEEE Internet Things J..

[10] Wang Y., Wu Z., Ni D. (2024). Real-time optimization of heliostat field aiming strategy via an improved swarm intelligence algorithm. Appl. Sci..

[11] Han M., Du Z., Yuen K.F., Zhu H., Li Y., Yuan Q. (2024). Walrus optimizer: A novel nature-inspired metaheuristic algorithm. Expert Syst. Appl..

[12] Fu S., Li K., Huang H., Ma C., Fan Q., Zhu Y. (2024). Red-billed blue magpie optimizer: A novel metaheuristic algorithm for 2D/3D UAV path planning and engineering design problems. Artif. Intell. Rev..

[13] El-Fergany A.A., Agwa A.M. (2024). Red-billed blue magpie optimizer for electrical characterization of fuel cells with prioritizing estimated parameters. Technologies.

[14] Ye M., Wang X., Guo Z., Hu B., Wang L. (2025). A multi-strategy improved red-billed blue magpie optimizer for global optimization. Biomimetics.

[15] Lu B., Xie Z., Wei J., Gu Y., Yan Y., Li Z., Zhou R. (2025). MRBMO: An enhanced red-billed blue magpie optimization algorithm for solving numerical optimization challenges. Symmetry.

[16] Fathy A., Agwa A.M. (2025). Red-Billed Blue Magpie Optimizer for Modeling and Estimating the State of Charge of Lithium-Ion Battery. Electrochem.

[17] Wang Q., Zhang M., Li L., Yan W., Liu B., Xia Z., Tseng M.L. (2025). Optimal capacity configuration of coupled photovoltaic and energy storage system: Multi-objective red-billed blue-magpie optimizer. J. Ind. Prod. Eng..

[18] Zhu C., Wang Z., Peng Y., Xiao W. (2025). An improved red-billed blue magpie feature selection algorithm for medical data processing. PLoS ONE.

[19] Zhang J., Li H., Zhang G., Chen R., Zhang T., Jin A. (2025). Red-billed blue magpie optimization for training feedforward neural networks. Sci. Rep..

[20] Adegboye O.R., Feda A.K., Kusetogullari H. (2026). Red-Billed Blue Magpie Optimization Algorithm-Based Aquila Optimizer: Numerical Optimization, Engineering Problem, and Cybersecurity Intrusion Prediction. Symmetry.

[21] Liu J., Huang X., Deng Y., Xiao C., Li Z. (2025). Robotic positioning accuracy enhancement via memory red billed blue magpie optimizer and adaptive momentum pso tuned graph neural network. Machines.

[22] Ibrahim A.W., Al-Shamma’a A.A., Xu J., Li D., Farh H.M.H., Alwesabi K. (2025). A Novel Red-Billed Blue Magpie Optimizer Tuned Adaptive Fractional-Order for Hybrid PV-TEG Systems Green Energy Harvesting-Based MPPT Algorithms. Fractal Fract..

[23] Kong W., Zhou M., Hu F., Zhu Z. (2025). Manuscript Title: Thermal-Electrical scheduling of Low-Carbon Industrial energy systems with rooftop PV: An improved Red-Billed blue magpie optimization approach. Therm. Sci. Eng. Prog..

[24] Ouyang K., Wei D., Fu S., Gu S., Sha X., Yu J., Yu J., Heidar A.A., Cai Z., Chen H. (2025). Multi-objective red-billed blue magpie optimizer: A novel algorithm for multi-objective UAV path planning. Results Eng..

[25] Fu Y., Liu D., Chen J., He L. (2024). Secretary bird optimization algorithm: A new metaheuristic for solving global optimization problems. Artif. Intell. Rev..

[26] Zhu D., Wang S., Zhou C., Yan S., Xue J. (2024). Human memory optimization algorithm: A memory-inspired optimizer for global optimization problems. Expert Syst. Appl..

[27] Cymerys K., Oszust M. (2024). Attraction–repulsion optimization algorithm for global optimization problems. Swarm Evol. Comput..

[28] Nurmuhammed M., Akdağ O., Karadağ T. (2024). Modified Archimedes optimization algorithm for global optimization problems: A comparative study. Neural Comput. Appl..

[29] Zhang L., Huang Z., Yang Z., Yang B., Yu S., Zhao S., Zhang X., Li X., Yang H., Lin Y. (2025). Tomato stem and leaf segmentation and phenotype parameter extraction based on improved red billed blue magpie optimization algorithm. Agriculture.

[30] Zheng C., Li X., You J., Liu Y., Ma X., Liu Y., Bai Y., Qiu X., Xiao B. (2025). Three-Dimensional Pendulum Red-Billed Blue Magpie Optimizer Hybrid Algorithm for Triaxial Magnetometer Calibration. IEEE Sens. J..

[31] Xue M., Zhu Y., Liu B., Song S., Zhang Z., Zhang S., Yu H., Wang L. (2025). ORBMO-RF: A non-destructive classification method for ginseng seeds based on multimodal fusion and improved red-billed blue magpie optimization algorithm. Front. Plant Sci..

[32] Zhang Z.L., Han C., Shen K., Hao Q., Jiang J., Zhang Z. (2024). Trajectory Tracking Control for USVs Based on Red-billed Blue Magpie Optimized ADRC. J. Phys. Conf. Ser..

[33] Almsallti M., Alzubi A.B., Adegboye O.R. (2025). Hybrid metaheuristic optimized extreme learning machine for sustainability focused co2 emission prediction using globalization-driven indicators. Sustainability.

[34] Janjarapu D.S.K., Venkata S.M., Pataiah S.K.A. (2025). Dual CH Selection and Routing for Network Lifetime Maximization in Wireless Sensor Network Using Red Billed Blue Magpie Optimization Algorithm. Int. J. Intell. Eng. Syst..

[35] Sun L., Gu M., Chen T., Liu X., Zheng H., Chen H. (2025). AR-RBMO: An enhanced red-billed blue magpie optimizer with attraction-repulsion and dynamic balancing strategies for global optimization. J. Comput. Des. Eng..

[36] Pereira J.L.J., Ma B.J., Francisco M.B., Junior R.F.R., Gomes G.F. (2023). A comparison between chaos theory and Lévy flights in sunflower optimization for feature selection. Expert Syst..

[37] Yan Y., Duan K., Cui J., Guo S., Cui C., Zhou Y., Huang J., Wang G., Zhang D., Zhang F. (2025). Nonlinear hysteresis parameter identification of piezoelectric actuators using an improved gray wolf optimizer with logistic chaos initialization and a levy flight variant. Micromachines.

[38] Alawida M. (2024). Enhancing logistic chaotic map for improved cryptographic security in random number generation. J. Inf. Secur. Appl..

[39] Xie X., Zhang K., Zheng B., Ning H., Zhou Y., Peng Q., Li Z. (2025). A CML-ECA Chaotic Image Encryption System Based on Multi-Source Perturbation Mechanism and Dynamic DNA Encoding. Symmetry.

[40] Alabed T., Servi S. (2025). A Lévy flight based chaotic black winged kite algorithm for solving optimization problems. Sci. Rep..

[41] Lai Y., Kang L., Cao W. (2025). A Multi-objective Particle Swarm Optimization using Logistics-tent Chaotic Map with GOBL and Non-inertial Lévy Flight. J. Comput..

[42] Mishra P., Ali M., Islam S. (2025). Enhanced mutation strategy based differential evolution for global optimization problems. PeerJ Comput. Sci..

[43] Maiti B., Biswas S., Ezugwu A.E.S., Bera U.K., Alzahrani A.I., Alblehai F., Abualigah L. (2025). Enhanced crayfish optimization algorithm with differential evolution’s mutation and crossover strategies for global optimization and engineering applications. Artif. Intell. Rev..

[44] Chauhan D., Suganthan P.N. (2025). Learning strategies for particle swarm optimizer: A critical review and performance analysis. Swarm Evol. Comput..

[45] Nadimi-Shahraki M.H., Zamani H., Asghari Varzaneh Z., Mirjalili S. (2023). A systematic review of the whale optimization algorithm: Theoretical foundation, improvements, and hybridizations. Arch. Comput. Methods Eng..

[46] Heidari A.A., Mirjalili S., Faris H., Aljarah I., Mafarja M., Chen H. (2019). Harris hawks optimization: Algorithm and applications. Future Gener. Comput. Syst..

[47] Xue J., Shen B. (2020). A novel swarm intelligence optimization approach: Sparrow search algorithm. Syst. Sci. Control Eng..

[48] Abualigah L., Diabat A. (2021). Advances in sine cosine algorithm: A comprehensive survey. Artif. Intell. Rev..

[49] Sahoo S.K., Saha A.K., Ezugwu A.E., Agushaka J.O., Abuhaija B., Alsoud A.R., Abualigah L. (2023). Moth flame optimization: Theory, modifications, hybridizations, and applications. Arch. Comput. Methods Eng..

[50] Xue J., Shen B. (2023). Dung beetle optimizer: A new meta-heuristic algorithm for global optimization. J. Supercomput..

[51] Hashim F.A., Houssein E.H., Hussain K., Mabrouk M.S., Al-Atabany W. (2022). Honey Badger Algorithm: New metaheuristic algorithm for solving optimization problems. Math. Comput. Simul..

